# Supplementary material for: Integrative reconstruction of cancer genome karyotypes using InfoGenomeR
Source: Nat Commun. 2021 Apr 29;12:2467. doi: 10.1038/s41467-021-22671-6 (PMC8085216; doi:10.1038/s41467-021-22671-6)
Supplement: Supplementary file 1 — Supplementary Information [file 41467_2021_22671_MOESM1_ESM.pdf]

Supplementary Information for Integrative reconstruction of cancer genome karyotypes using InfoGenomeR

Yeonghun Lee[1] & Hyunju Lee[1]

[1] *School of Electrical Engineering and Computer Science, Gwangju Institute of Science and Technology, Gwangju 61005, South Korea.*

Contents

Supplementary Notes **4**

1 Cancer genome simulation 4

2 Analysis of WGS data from the HeLa cell line 5

3 Complex structural variations (SVs) and SV clusters 6

4 Characterisation of complex SVs in cancers 9

5 Finding SV edges by remapping nonproperly paired (NP) reads in breakpoint graph construction 10

6 Breakpoint graph construction for somatic SVs 12

7 Classification of simple SVs based on the breakpoint graph 13

8 Breakpoint graph simplification 14

9 Expectation-maximisation (EM) algorithm for the estimation of allele-specific copy number model parameters 15

10 Optimisation for the allele-specific breakpoint graph 16

11 Nonhomologous SVs and haplotype segments 19

12 Haplotype phasing 22

13 Multiway tree for enumeration of Eulerian paths 23

14 Breakpoint graph construction for multi-sample data 24

Supplementary Figures **25**

Supplementary Tables **50**

Supplementary References **63**

Supplementary Note 1: Cancer genome simulation.

First, we generated 12 simulated germline genomes from the NA12878, HG00732, NA19238, and HG00513 individuals, which are common benchmarks for structural variations (SVs), in phase 3 of the 1000 Genomes Project[1,2]. All the SNPs were retained to reflect the density of SNPs and linkage disequilibrium for allele-specific and haplotype estimation. For germline SVs (deletions, tandem duplications, inversions, and insertions), we simulated 3000 germline SVs per germline genome by randomly selecting 1500 germline SVs from the reported SVs in phase 3 individuals and randomly generating 1500 germline SVs. The proportion and size of each generated SV type were derived from the phase 3 individuals, where the size of SVs was drawn from a beta distribution (Supplementary Table 6)[3]. As germline variants tend to occur in regions with low mappability[4], we generated 20% of the variants in such regions with a mappability score of <0.5 based on the UCSC mappability track.

Next, we generated a cancer genome from each germline genome by simulating approximately 200 somatic SVs (deletions, tandem duplications, inversions, insertions, and interchromosomal and intrachromosomal rearrangements) based on a previous study of 140 TCGA cancer genomes[5]. To simulate somatic SVs, we used 13 operations using known mechanisms (Supplementary Fig. 21). Tandem duplications, deletions, inversions, and insertions were generated randomly, the size of which was drawn from a beta distribution (Supplementary Table 6). For complex SVs, we simulated breakage-fusion-bridge (BFB) cycles, chromothripsis, and template switching, where SV breakpoints were randomly assigned across chromosomes. Chromosomal or arm-level amplification, deletion, and whole-genome duplication were used to generate CNAs and aneuploidy, up to

tetraploidy. The total proportion of somatic SV types was derived from a previous study (Supplementary Table 6)[5]. For each cancer genome, cancer purity was simulated at 60%, 75%, and 90%, which were previously reported as the lowest, average, and highest purities of TCGA cancer types, respectively [6]. Each cancer genome was mixed with the matched germline genome to generate a mixed population according to cancer purity. We simulated Illumina HiSeq 2000 2x100 reads (350 bp insert) from heterogeneous genomes with 3X, 5X, 10X, 15X, and 20X haplotype fold coverages using ART (version 2.5.8)[7], and reads were mapped to the GRCh37 reference genome using BWA-MEM (version 0.7.15)[8].

To compare performance depending on the human reference genome versions, we generated three additional cancer genomes from the NA12878 based on GRCh38. SNP and SV positions were lifted over using the ucsc liftOver tool from phase 3 of the 1000 Genomes Project. All the simulation schemes were same as those used in the GRCh37 simulation, and reads were mapped to the GRCh38 reference genome using BWA-MEM.

Supplementary Note 2: Analysis of WGS data from the HeLa cell line.

We applied InfoGenomeR to WGS data from the HeLa cell line[9] to reconstruct the HeLa karyotype, an application involving real data with substantial SV noise. We detected 19,381 initial SVs using DELLY2, Manta, and novoBreak, including 9,289 initial translocation calls. InfoGenomeR performed iterative optimisation and selected 1,736 SVs from the initial SV calls for initial breakpoint graph construction (Supplementary Fig. 8a). Based on the breakpoint graph, we classified 746 small (<100 kb), simple SVs (insertions, duplications, deletions, and inversions) from 1,736

SV calls. These SVs included small germline events that had insufficient allelic information and therefore they were excluded from subsequent analyses. After removing the small simple SVs and re-optimising the breakpoint graph (Supplementary Fig. 8b), InfoGenomeR finally obtained 94 SVs (22 deletions, 32 duplications, 31 intrachromosomal, and 9 interchromosomal rearrangements) with a 0.97 cancer purity and 3.04 ploidy. Based on the 94 SVs detected by InfoGenomeR, we constructed a haplotype graph for the HeLa cell line. The allelic measurement resulted in an overall 1:2 allele ratio, where arm-level or whole-chromosome CNAs or losses of heterozygosity (LOHs) were frequent. The haplotype graph included multiple karyotypic scenarios according to alternative Eulerian paths. After excluding simple deletions and duplications (<1 Mb), which exponentially increased Eulerian paths and were ignored in the karyotype analysis, each set of chromosomes grouped using interchromosomal SVs had between one and 96 scenarios.

Supplementary Note 3: Complex SVs and SV clusters.

Complex SVs are reconstructed in the haplotype graph, and they include fold-back inversions, balanced translocations, and unbalanced translocations, which are representative of the karyotypic characteristics in cancer.

- Fold-back inversion (FB): A head-to-head SV or tail-to-tail SV (<1 Mb) with increase in copy number.
- Balanced translocation: Interchromosomal rearrangements exist at the same breakpoints (copy-neutral) or within <1 Mb deletion bridges[10].

- Unbalanced translocation: The other interchromosomal rearrangements are classified into unbalanced translocations, where a chromosomal loss or gain exists.

Considerable parts of complex SVs are clustered with each other in a focal manner, and they need to be considered as a unified set to characterise them in cancer genome structures. Therefore, we define an SV cluster, an inductively defined set of SV edges found in a breadth-first search manner, as: 1) $e_v \in$ a SV cluster 2) if a simple tour of <5 Mb exists between e_v and e'_v, then, $e'_v \in$ the SV cluster. A tour in the breakpoint graph alternates between the SV edges/reference edges and segment edges. If the tour crosses an SV edge, it comes to an adjacent segment edge and leaves an adjacent SV or reference edge. The simple tour indicates a tour composed of reference and segment edges without SV edges. We find SV clusters with >4 SV edges. Then, we classify SV clusters into three amplifications types, namely, HSR, HSR/DM, and DM, and a deletion type, CT. These are the definitions of the terms used for SV clusters in the study.

- HSR: One of the flanked genomic segments in the SV cluster is highly amplified (>10 copies), and the SV cluster is connected to a chromosomal arm. This means that the amplified segment is crossed more than 10 times in the tour of the SV cluster and ends at the chromosomal arm, which has been commonly described as an HSR in cytogenetic analyses.
- HSR/DM: The cluster satisfies the HSR condition, and a cycle with at least five multiplicities exists in the SV cluster from the minimum entropy search. DM is a small circular segment capable of autonomously replicating up to hundreds of copies, where

heterogeneity of DM structures could exist[11]. The coexistence of an HSR and a DM is commonly observed and explained by the episome model in which a DM is excised and replicated, and some of them are incorporated into a chromosome to generate HSR[12]. Although a sample has only DMs, it could be observed as an HSR/DM if the other intact homologous chromosome was present. In this case, a reference edge was found between the DMs and adjacent genomic segments (from the intact chromosome). Therefore, we could not strictly define DMs. An HSR/DM represents the ambiguity between an HSR and a DM from WGS data or true coexistence at the same time.

- DM: A cycle with at least five multiplicities exists in the SV cluster from the minimum entropy search without being connected to a chromosomal arm. No reference edge is found between the cycle and the chromosomal arm, and cycles exist extrachromosomally.

- CT: Canonical chromothripsis is defined based on the criteria from other studies[13,14]. SV clusters that we obtained from the haplotype graph can detect conditions satisfying the clustering of breakpoints, ability to walk the derivative chromosome, and prevalence of rearrangements affecting a specific haplotype. For interspersed LOH, we set the minimum number of interspersed LOHs to three. For randomness of DNA segment order and fragment joins, we performed a chi-square test for the randomness of SV orientations (HH, HT, TH, and TT) and rejected an SV cluster with a p-value <0.01. Regularity of oscillating copy-number states is restricted to cases with deletions, and it is violated when other complex SVs causing amplifications are involved with CT[14]. We

observed that several cases had chromothripsis patterns along with other amplification types (HSR, HSR/DM, and DM), and we made exclusions in the criteria for them[14].

Supplementary Note 4: Characterisation of complex SVs in cancers.

We applied InfoGenomeR to TCGA datasets of breast invasive carcinoma (BRCA, n=90), glioblastoma multiforme (GBM, n=37), and ovarian serous cystadenocarcinoma (OV, n=47). The average numbers of somatic SVs were 223, 124, and 275, respectively, more than 20% of which were complex SVs that were not classified into simple SVs (deletions, tandem duplications, inversions, and insertions) (Supplementary Fig. 11a). We found that these complex SVs were clustered with each other in the breakpoint graph rearranging focal segments (<5Mb), resulting in SV clusters, which may indicate an amplification origin of oncogenes such as HSR and DM, as shown in karyotyping analyses[15]. The average percentages of clustered SVs among complex SVs were 36%, 44%, and 16% in BRCAs, GBMs, and OVs, respectively, which were consistent with frequent observations of HSRs or DMs in BRCAs and GBMs through traditional karyotyping[15]. Each sample had up to six SV clusters, each of which contained 18 complex SVs on average (5 to 126) (Supplementary Fig. 11a).

Focal segments in SV clusters were highly amplified (mean 15 copies) encompassing oncogenes in concordance with GISTIC peaks (https://gdac.broadinstitute.org/). Hotspots of SV clusters were on chromosomes 1, 8, 11, and 17 in BRCAs, chromosomes 1, 4, 7, and 12 in GBMs, and chromosomes 3, 5, and 19 in OVs, respectively (Supplementary Fig. 11b). Oncogenes were commonly amplified in SV clusters rather than in simple duplications (Supplementary Fig. 11c), suggesting

that these SV clusters were the hotspots of oncogene amplification. Among the SV clusters, *ERBB2* has the highest amplification rate (79.2%), followed by *CCND1* (64.7%) and *PPM1D* (50.0%) in BRCAs; *EGFR* (52.1%), *CDK4* (61.5%), *MDM2* (75.0%), and *KIT* (33.3%) in GBMs; and *BRD4* (35.3%) and *CCNE1* (18.8%) in OVs, respectively.

Supplementary Note 5: Finding SV edges by remapping nonproperly paired (NP) reads in breakpoint graph construction.

We performed the first-round iterations for breakpoint graph construction and then added putative SV edges, which could either be misfiltered during the first-round iterations or missing from the initial SV callings. The former results from oversegmentation with numerous false-positive SVs, and SVs could be incorrectly filtered due to low-confident copy numbers (CNs) by oversegmentation. The latter results from read mapping errors or SV-calling errors from mapped data (BAM), where the paired-end read information of true SVs is hidden[16]. To rescue these SVs, we first extracted NP reads from the initial BAM file using SAMtools with the -F 2 option and searched for read pairs derived from candidate adjacencies from unbalanced nodes in the breakpoint graph. To find NP read pairs from candidate adjacencies, we remapped NP reads using BWA-MEM with the -h option (multiple alignments) to reference sequences encompassing the +/-50000 bp downstream and upstream of the unbalanced nodes. The offsets are used to correct the inaccurate breakpoints of unbalanced nodes, which will be adjusted using the SV breakpoints that we determine as following steps. We selected candidate adjacencies with >5 supporting NP read pairs.

Next, we find confident NP read pairs by measuring the aspect of mapping quality[17]. Note that

NP reads supporting the candidate adjacency, which we found in the previous step, could come from other reference sequences similar to the candidate adjacency. We used BLAT (version 36)[18] to search for possible mapping positions of NP reads, which enables more precise read mapping, compared to BWA-MEM. The mapping quality of the read that comes from the genomic region can be estimated from the possible mapping positions, M, that we searched using BLAT. The probability $p(z|g,u)$ that a read z is sequenced from a position u in a reference genome g is the product of mismatched error probabilities compared to the reference genome[17]. The mismatched error probability is 10^{-q}, where q is the phred base quality. Q denotes a phred base quality set of all mismatches compared to the reference sequence.

$$p(z|g,u) = \prod_{q \in Q} (10^{-q}) \tag{1}$$

$$p(u|g,z) = \frac{p(z|g,u)}{\sum_{m \in M} p(z|g,m)} \tag{2}$$

The mapping quality for paired reads, $p(u_1, u_2|g, z)$, is the product of the probabilities of individual reads of the read pair, assuming independence between them. In addition, we obtained SNVs from the mapped data, which are regarded as true variants in a cancer sample, so g is replaced with a genotyped sequence with SNVs, and the mapping quality is recalibrated. Then, we count the number of NP read pairs that support the SV edge with a mapping quality $p(u_1, u_2|g, z) >$0.9. The breakpoint graph gives the multiplicities of candidate SV edges from unbalanced nodes, which is the minimum among the remaining CNs of unbalanced nodes. When the multiplicity μ of a candidate SV edge is given, the number of NP read pairs, n, which comes from the SV edge, can be modelled as a Poisson process. Here, λ is the haplotype base coverage, which we approximate as the base coverage divided by the mean ploidy obtained from the purity and ploidy estimation

(iterative step 2 in the Methods section in the main document).

$$P(n|\mu) = \frac{(\mu\lambda)^n}{n!} e^{-\mu\lambda} \tag{3}$$

We remove candidate adjacencies using $P(n \leq N|\mu) < 0.01$, and the others remain in the breakpoint graph. The candidate adjacencies go through the second-round iterations that stabilise the breakpoint graph with the low-segmentation parameter, and the breakpoint graph is finalised through filtering false SVs again to reduce the objective function.

Supplementary Note 6: Breakpoint graph construction for somatic SVs.

Cancer genomes are characterised by somatic SVs, and the germline SVs can be excluded if a control (normal) genome exists. For somatic SV breakpoint graph construction, we measured somatic CNAs by normalising read counts of cancer genomes through reading counts of normal genomes using BIC-seq2. Somatic CNA measurements require the subtraction of genomic bins in putative germline variant regions, as these are obstacles for the CNA measurements which assume that the control segments are diploid (two copies). For this requirement, first, InfoGenomeR reconstructs the control genome using germline variants in the same way as in the breakpoint graph construction. Next, copy number bins in germline variants are excluded from the cancer genome, and we perform breakpoint graph construction with somatic SVs using copy number bins of the regions where the control genome has no variants.

Supplementary Note 7: Classification of simple SVs based on the breakpoint graph.

Based on the breakpoint graph, we classify SVs as simple SVs (tandem duplications, deletions, inversions, or block-interchange insertions) and complex SVs (other intrachromosomal or interchromosomal rearrangements). Simple SVs are defined as 1) SVs shorter than 1 Mb, 2) no additional breakpoints in the genomic segment flanked by SVs, and 3) belonging to the following categories (Supplementary Fig. 17a). Note that head-to-head, head-to-tail, tail-to-head, and tail-to-tail notations are assigned based on the orientation of the breakpoint adjacencies to the genomic segments in the coordinate order of the reference sequence.

- Tandem duplication (TD): The segment is flanked by a head-to-tail SV with an increase in copy number.
- Deletion (D): The segment is flanked by a tail-to-head SV with a decrease in copy number.
- Block-interchange insertions (INS): The segment is flanked by SVs and is inserted into other chromosomal regions (copy-neutral and copy-deleted insertions).
- Inversions (INV): The segment is flanked by head-to-head and tail-to-tail SVs (copy-neutral and copy-deleted inversions).

Complex SV types include fold-back inversion (FB), balanced and unbalanced translocation (T), and other complex rearrangements such as homogeneously staining regions (HSRs), homogeneously staining region/double minutes (HSR/DMs), double minutes (DMs), and chromothripsis

(CT). Their classifications require a haplotype-level karyotypic configuration, which we address after haplotype graph construction (see the Complex SVs and SV clusters section).

Supplementary Note 8: Breakpoint graph simplification.

Short simple SVs (<100 kb) are negligible in a karyotypic scope, and the number of heterozygous SNPs in the short segment could be insufficient to provide confident allelic ratios, which are important for allele-specific graph construction. Therefore, after breakpoint graph construction, we exclude short simple SVs, classified as tandem duplications, deletions, insertions, or inversions, and then subtract copy number bins in short SVs. Removing these SVs and copy number bins affects few Eulerian paths in a karyotypic scope, while still maintaining the path orientation. In addition, short simple SVs caused an oversegmentation of the genome that prevented an accurate measurement of multiplicities of segment edges across the genome. For instance, the true measurement of segment edges for the ABC and ABBCx2 triploid genomes is $\mu(A) = 3$, $\mu(B) = 5$, and $\mu(C) = 3$, respectively. However, an error in the segment edge multiplicity for C could arise as $\mu(C) = 4$. By removing the B segment and representing the segment as A[removed B]C, we could measure $\mu(A[\text{removed } B]C) = 3$ accurately, thereby lowering the objective function for the copy number balance condition. Here, we replace the ABC and ABBCx2 genomes with the ABCx3 genome for the simplification process. The simplification requires additional iterative steps until the breakpoint graph converges after removing the SV edges and copy number bins for simple SVs.

Supplementary Note 9: Expectation-maximisation (EM) algorithm for the estimation of allele-specific copy number model parameters.

The integer copy number of the segment can be divided into a pair of ASCNs, $A_i = \{A_{i,1}, A_{i,2}\}$, where i denotes an index for candidate ASCN pairs, and each jth heterozygous SNP depth in the segment, $(o_{j,1}, o_{j,2})$, can be observed from $(A_{i,1}, A_{i,2})$, or $(A_{i,2}, A_{i,1})$. We use the latent variable $a_j = (a_{j,1}, a_{j,2})$ to indicate that $o_{j,1}$ is observed from $a_{j,1}$, and $o_{j,2}$ is observed from $a_{j,2}$ in order, and there are two possibilities: $a_j = (A_{i,1}, A_{i,2})$ or $(A_{i,2}, A_{i,1})$. When a_j is given per SNP, we can model o_j using a negative binomial distribution.

$$p(o_j|a_j, b, p, \phi_1, \phi_2) = \text{NB}(o_{j,1}|b(pa_{j,1} + (1-p)), \phi_{j,1})\text{NB}(o_{j,2}|b(pa_{j,2} + (1-p)), \phi_{j,2}) \quad (4)$$

Our goal is to find parameters through maximising the likelihood function, which is the product of the sum of the joint distribution over latent variables.

$$p(O|\Theta) = \prod_{j=1}^{N} \sum_{a_j} p(o_j, a_j|b, p, \phi_1, \phi_2) \quad (5)$$

Instead of directly solving for the maximum log-likelihood, the EM algorithm estimates model parameters with latent variables by iteratively updating Θ in the expectation and maximisation steps, where $\Theta = \{b, \phi_1, \phi_2\}$ and p are estimated using ABSOLUTE (iterative step 2 in the Methods section of the main paper).

Expectation step: Given Θ^{old}, we compute the conditional distribution of a_j using Bayes' theorem. We assume that the prior probabilities of a_j, $p(a_j = (A_{i,1}, A_{i,2}))$, and $p(a_j = (A_{i,2}, A_{i,1}))$, are the

same because the order of ASCNs for each heterozygous SNP is randomly observed.

$$p(a_j = (A_{i,1}, A_{i,2})|o_j, \Theta^{\text{old}}) = \frac{p(o_j|a_j = (A_{i,1}, A_{i,2}), \Theta^{\text{old}})p(a_j = (A_{i,1}, A_{i,2}))}{p(o_j|a_j = (A_{i,2}, A_{i,1}), \Theta^{\text{old}})p(a_j = (A_{i,2}, A_{i,1})) + p(o_j|a_j = (A_{i,1}, A_{i,2}), \Theta^{\text{old}})p(a_j = (A_{i,1}, A_{i,2}))} \tag{6}$$

Next, we compute $Q(\Theta|\Theta^{\text{old}})$, which is the expectation of the log likelihood of Θ with respect to the conditional distribution of a_j, given o_j and Θ^{old}.

$$Q(\Theta|\Theta^{\text{old}}) = \sum_{j=1}^{N} \sum_{a_j} p(a_j|o_j, \Theta^{\text{old}})\log(p(a_j, o_j|\Theta)) \tag{7}$$

Maximisation step: We find a new Θ that maximises $Q(\Theta|\Theta^{\text{old}})$.

Supplementary Note 10: Optimisation for the allele-specific breakpoint graph.

The ASCN measurement of each segment can be balanced or imbalanced, and if it is imbalanced, the segment is divided into two allelic segments in the allele-specific graph. If the ASCN measurement of the segment is low-confidence, its multiplicities are not fixed and instead determined using the search for the best ASCNs that minimise the objective function. To find multiplicities minimising the objective function, we first solve the integer programming problem in the series of imbalanced AS segments locally.

We denote the head and tail allelic nodes of the ith imbalanced segment as $s^i_{1,h}$ and $s^i_{1,t}$ for allele 1, and $s^i_{2,h}$ and $s^i_{2,t}$ for allele 2, respectively. The original objective function for the nodes between the ith and $i+1$th segments includes the differences between the right-hand sum and the left-hand

sum of the copy number balance condition.

$$\begin{aligned} &\mu(e_s(s^i_{1,t})) - \mu(e_r(s^i_{1,t})) - \sum_{v^i_{1,t} \in E_v(s^i_{1,t})} \mu(v^i_{1,t}) \\ &+\mu(e_s(s^{i+1}_{1,h})) - \mu(e_r(s^{i+1}_{1,h})) - \sum_{v^{i+1}_{1,h} \in E_v(s^{i+1}_{1,h})} \mu(v^{i+1}_{1,h}) \\ &+\mu(e_s(s^i_{2,t})) - \mu(e_r(s^i_{2,t})) - \sum_{v^i_{2,t} \in E_v(s^i_{2,t})} \mu(v^i_{2,t}) \\ &+\mu(e_s(s^{i+1}_{2,h})) - \mu(e_r(s^{i+1}_{2,h})) - \sum_{v^{i+1}_{2,h} \in E_v(s^{i+1}_{2,h})} \mu(v^{i+1}_{2,h}) \end{aligned} \tag{8}$$

The second constraint in the integer programming problem of the allele-specific breakpoint graph construction section in our study is that the multiplicities of SV edges cannot exceed the difference between multiplicities of the ith and $i+1$th segment edges. In other words, the multiplicity of the reference edge between the ith and $i+1$th segments is maximised to minimise the objective function when the second constraint is given. The multiplicity of the reference edge between the ith allele 1 segment and $i+1$th allele 1 segment, $\mu(e_r(s^i_{1,t})) = \mu(e_r(s^{i+1}_{1,h}))$, is temporarily chosen by $\min(\mu(e_s(s^i_{1,t})), \mu(e_s(s^{i+1}_{1,h})))$. For allele 2, $\mu(e_r(s^i_{2,t})) = \mu(e_r(s^{i+1}_{2,h}))$, is chosen as $\min(\mu(e_s(s^i_{2,t})), \mu(e_s(s^{i+1}_{2,h})))$. Note that when two SV edges are exactly reciprocal, a zero-length (low-confidence) segment between reciprocal breakpoints is added to prevent the second constraint from removing the exact reciprocal events. The objective function is simplified as follows:

$$\begin{aligned} &\mu(e_s(s^i_{1,t})) + \mu(e_s(s^{i+1}_{1,h})) - 2 \times \min(\mu(e_s(s^i_{1,t})), \mu(e_s(e^{i+1}_{1,h}))) - \sum_{v^i_{1,t} \in E_v(s^i_{1,t})} \mu(v^i_{1,t}) - \sum_{v^{i+1}_{1,h} \in E_v(s^{i+1}_{1,h})} \mu(v^{i+1}_{1,h}) \\ &+\mu(e_s(s^i_{2,t})) + \mu(e_s(s^{i+1}_{2,h})) - 2 \times \min(\mu(e_s(s^i_{2,t})), \mu(e_s(s^{i+1}_{2,h}))) - \sum_{v^i_{2,t} \in E_v(s^i_{2,t})} \mu(v^i_{2,t}) - \sum_{v^{i+1}_{2,h} \in E_v(s^{i+1}_{2,h})} \mu(v^{i+1}_{2,h})) \end{aligned} \tag{9}$$

The objective function can be simplified further using the equation $A + B - 2 \times \min(A, B) = |A - B|$.

$$
\begin{aligned}
&|\mu(e_s(s^i_{1,t})) - \mu(e_s(s^{i+1}_{1,h}))| - \sum_{v^i_{1,t} \in E_v(s^i_{1,t})} \mu(v^i_{1,t}) - \sum_{v^{i+1}_{1,h} \in E_v(s^{i+1}_{1,h})} \mu(v^{i+1}_{1,h}) \\
+&|\mu(e_s(s^i_{2,t})) - \mu(e_s(s^{i+1}_{2,h}))| - \sum_{v^i_{2,t} \in E_v(s^i_{2,t})} \mu(v^i_{2,t}) - \sum_{v^{i+1}_{2,h} \in E_v(s^{i+1}_{2,h})} \mu(v^{i+1}_{2,h})
\end{aligned}
\tag{10}
$$

The multiplicity of the SV edge, v^i, depends on both the ith segment (breakpoint 1) and the other segment (breakpoint 2) to which the SV edge is connected, but we temporarily select $\mu(v^i)$, independent of breakpoint 2, as a greedy choice. If the breakpoint 2 is at the $i - j$th position that we previously processed, multiplicity is chosen depending on both breakpoint 1 and breakpoint 2. In addition, if v^i is somatic (defined as SVs >1 Mb if a control does not exists), we assume that the same breakpoint does not occur in both alleles and that either $\mu(v^i_1)$ or $\mu(v^i_2)$ is zero (infinite site assumption)[19,20]. The greedy optimisation allows a linear computing time in each series of imbalanced AS segments.

After solving the integer programming problem for imbalanced AS segments, we assign the multiplicities of low-confidence segments that require perturbations to find the solution satisfying the copy number balance condition. We defined low-confidence ASCNs as $\text{Score}_L(\hat{A}) < 0.8$ or <5 heterozygous SNPs. For segments of node s with $\text{Score}_L(\hat{A}) < 0.8$ or <5 heterozygous SNPs, we search for the best ASCNs with a penalty (+1, +2, +3, ...), $\varepsilon(s)$, according to the rank of $\text{Score}_L(A)$, which is added to the objective function, preventing a solution far away from the initial estimate (Supplementary Fig. 18). For segments with no ASCN estimation, the initial estimates of ASCNs are inherited from the adjacent ASCNs, and then we search for the best ASCNs by changing them. We minimise the objective function by alternately changing low-confidence ASCNs in the

interrelated subset of nodes S_{related}, which can be obtained in the same way as the breakpoint graph construction. The final objective function with low-confidence ASCNs contains penalty terms added to the previous copy number balance equation.

$$\text{Minimise} \sum_{s \in S_{\text{related}}} (\mu(e_s(s)) - \mu(e_r(s)) - \sum_{v \in E_v(s)} \mu(v) + \varepsilon(s)/2) \tag{11}$$

Supplementary Note 11: Nonhomologous SVs and haplotype segments.

SVs can occur through nonallelic homologous recombination (NAHR) or other mechanisms that do not require homologous sequences (>100 bp homology), such as nonhomologous end joining (NHEJ) and replication-based mechanisms (RBMs)[21]. NAHR results from crossovers that exchange allelic information (interhomolog) or from sister chromatid exchanges (intrahomolog), and they are prevalent in germline cells, whereas NHEJ and RBMs occur frequently in somatic events, explaining the complex recombinations observed in cancers that are not mediated by homologous sequences[5]. NHEJ generates SVs during the process of double-strand break (DSB) repair, and RBMs generate SVs through replication slippage in a replication fork or fork stalling and template switching (FoSTeS) in adjacent replication forks.

We performed a local alignment between SV breakpoints using BLAST[22] and classified SVs with a percent identity>90% in more than 100-bp homologous sequences as homologous recombinations[5] and the others as nonhomologous SVs (Supplementary Fig. 19a). Here, we assume that nonhomologous SVs (<1 Mb) occur in a single allele rather than between homologous chromosomes and perform prephasing of ASCNs. We exclude rare possibilities in which nonhomologous SVs are generated from simultaneous DSBs or replication fork collapses between homologous chromo-

somes within the same focal region (<1 Mb). Instead, these SVs are assumed to be generated from a single DSB or replication slippage event (simple SVs such as tandem duplications and deletions) or adjacent replication fork collapses in a homolog. This assumption simplifies the haplotype phasing problem, preventing unlikely allelic switches between the focal regions.

The haplotype segment $H_i = \{H_{i,1}, H_{i,2}\}$ is a set of sequences of imbalanced nodes, where a head and tail node pair define a segment and focal nonhomologous SVs (<1 Mb) are found between them. For instance, if there are two sequences of imbalanced segments, the $k, k+1, ..., k+k'$th segments and the $l, l+1, ..., l+l'$th segments, in the allele-specific graph, then $H_{i,1}$ and $H_{i,2}$ are defined as follows:

$$\begin{aligned} H_{i,1} &= \{(h^k_{1,h}, h^k_{1,t}), ..., (h^{k+k'}_{1,h}, h^{k+k'}_{1,t}), (h^l_{1,h}, h^l_{1,t}), ..., (h^{l+l'}_{1,h}, h^{l+l'}_{1,t})\} \\ H_{i,2} &= \{(h^k_{2,h}, h^k_{2,t}), ..., (h^{k+k'}_{2,h}, h^{k+k'}_{2,t}), (h^l_{2,h}, h^l_{2,t}), ..., (h^{l+l'}_{2,h}, h^{l+l'}_{2,t})\} \end{aligned} \tag{12}$$

There are two possibilities from the allele-specific graph.

$$\begin{aligned} H_{i,1} &= \{(s^k_{1,h}, s^k_{1,t}), ..., (s^{k+k'}_{1,h}, s^{k+k'}_{1,t}), (s^l_{1,h}, s^l_{1,t}), ..., (s^{l+l'}_{1,h}, s^{l+l'}_{1,t})\} \\ H_{i,2} &= \{(s^k_{2,h}, s^k_{2,t}), ..., (s^{k+k'}_{2,h}, s^{k+k'}_{2,t}), (s^l_{2,h}, s^l_{2,t}), ..., (s^{l+l'}_{2,h}, s^{l+l'}_{2,t})\} \\ &\text{or} \\ H_{i,1} &= \{(s^k_{1,h}, s^k_{1,t}), ..., (s^{k+k'}_{1,h}, s^{k+k'}_{1,t}), (s^l_{2,h}, s^l_{2,t}), ..., (s^{l+l'}_{2,h}, s^{l+l'}_{2,t})\} \\ H_{i,2} &= \{(s^k_{2,h}, s^k_{2,t}), ..., (s^{k+k'}_{2,h}, s^{k+k'}_{2,t}), (s^l_{1,h}, s^l_{1,t}), ..., (s^{l+l'}_{1,h}, s^{l+l'}_{1,t})\} \end{aligned} \tag{13}$$

The allelic segment is defined by the pair of head and tail allelic nodes. The union of interrelated node sets, $H_{\text{related}} = H^k_{\text{related}} \cup H^l_{\text{related}}$, contains adjacent nodes together with imbalanced nodes in the haplotype segment. Here, the k, $k+1$, ..., $k+k'$th sequences of imbalanced segments are surrounded by balanced nodes, s^{k-1}_t and $s^{k+k'+1}_h$, and we temporarily divide the balanced nodes,

s_t^{k-1} and $s_h^{k+k'+1}$ into $h_{1,t}^{k-1}$ and $h_{2,t}^{k-1}$, and $h_{1,h}^{k+k'+1}$ and $h_{2,t}^{k+k'+1}$, respectively. The multiplicities are equal, $\mu(h_{1,t}^{k-1}) = \mu(h_{2,t}^{k-1})$ and $\mu(h_{1,h}^{k+k'+1}) = \mu(h_{2,h}^{k+k'+1})$.

$$H_{\text{related}}^k = \{h_{1,t}^{k-1}, h_{2,t}^{k-1}\} \cup \{h_{1,h}^k, h_{1,t}^k, ..., h_{1,h}^{k+k'}, h_{1,t}^{k+k'}\} \cup \{h_{2,h}^k, h_{2,t}^k, ..., h_{2,h}^{k+k'}, h_{2,t}^{k+k'}\} \cup \{h_{1,h}^{k+k'+1}, h_{2,h}^{k+k'+1}\}$$
$$H_{\text{related}}^l = \{h_{1,t}^{l-1}, h_{2,t}^{l-1}\} \cup \{h_{1,h}^l, h_{1,t}^l, ..., h_{1,h}^{l+l'}, h_{1,t}^{l+l'}\} \cup \{h_{2,h}^l, h_{2,t}^l, ..., h_{2,h}^{l+l'}, h_{2,t}^{l+l'}\} \cup \{h_{1,h}^{l+l'+1}, h_{2,h}^{l+l'+1}\} \quad (14)$$

We select the order between the $k, k+1, ...$ segments and the $l, l+1, ...$ segments by minimising the objective function between them using the constraint for nonhomologous SVs. For instance, a nonhomologous SV, nv, has two breakpoints, nv1 and nv2, and hap(nv1) and hap(nv2) indicate the haplotype (1 or 2, respectively) in which the breakpoints occur. We assume that nv occurs in the same haplotype and exclude the case in which hap(nv1) $\neq$ hap(nv2) occurs, by adding it as an additional constraint.

$$\text{Minimise} \sum_{h \in H_{\text{related}}} (\mu(e_s(h)) - \mu(e_r(h)) - \sum_{\text{nv} \in E_{\text{nv}}(h)} \mu(\text{nv})) \quad (15)$$
$$\text{subject to}$$
$$\mu(\text{nv}) = 0 \text{ if hap(nv1)} \neq \text{hap(nv2)}$$

When haplotype segments are defined, the heterozygous SNPs $\text{snp}_{j,1}$ and $\text{snp}_{j,2}$ are phased according to SNP depths $o_{j,1}$ and $o_{j,2}$, respectively, using maximum likelihood estimation. Let us assume that the jth SNPs are from the kth segment in the haplotype segment $H_i = \{H_{i,1}, H_{i,2}\}$. We phase

heterozygous SNPs to add them to the SNP set of each haplotype segment, $H_i^{\text{snp}} = \{H_{i,1}^{\text{snp}}, H_{i,2}^{\text{snp}}\}$.

$$\text{snp}_{j,1} \in H_{i,1}^{\text{snp}} \text{ and } \text{snp}_{j,2} \in H_{i,2}^{\text{snp}} \text{ if}$$

$$p((o_{j,1}, o_{j,2})|(\mu(e_s(h_{1,h}^k)), \mu(e_s(h_{2,h}^k))), \Theta_k) > p((o_{j,2}, o_{j,1})|(\mu(e_s(h_{1,h}^k)), \mu(e_s(h_{2,h}^k))), \Theta_k), \quad (16)$$

$$\text{or vice versa.}$$

Supplementary Note 12: Haplotype phasing.

Haplotype phasing was performed using the hidden Markov model (HMM) of BEAGLE[23], where transition and emission probabilities are defined based on a localised haplotype-cluster graph. The localised haplotype-cluster graph was obtained from https://faculty.washington.edu/browning/beagle/b4_1.html, which was constructed from the 1000 Genomes Project phase 3 reference panel.

In brief, each edge e represents an allele and the transition probabilities $P(e_{j+1}|e_j) = n(e_{j+1})/n_p(e_{j+1})$, where $n(e)$ denotes the number of samples with the allele, and $n_p(e)$ denotes the total number of samples with edges of the previous allele, which are adjacent to e in the haplotype-cluster graph. In a diploid, the transition probabilities $P(e_{j+1}|e_j) = P((e_{1,j+1}, e_{2,j+1})|(e_{1,j}, e_{2,j})) = (n(e_{1,j+1})/n_p(e_{1,j+1}))(n(e_{2,j+1})/n_p(e_{2,j+1}))$ are the products of each transition probability. The emission probability is 1 if the observed alleles g are matched with the alleles of edges.

To obtain the most likely haplotype given the partial hidden states (heterozygous SNPs from haplotype segments); we used a constrained version of the Viterbi algorithm, where the Viterbi path was enforced to follow given hidden states (Supplementary Fig. 19b). For instance, the sets of heterozygous SNPs from the ith haplotype segment, $H_{i,1}^{\text{snp}}$ and $H_{i,2}^{\text{snp}}$, restrict the Viterbi path from the $p - q$th heterozygous alleles to the pth heterozygous alleles from the phase 3 panel. The dis-

tance q is the number of homozygous alleles between the $p-q$th and pth heterozygous alleles. The constrained Viterbi algorithm requires a local trace-back function, $T(e_p)^q$, which denotes the qth alleles back from the pth alleles, and it should be equal to the given alleles $\hat{e}_{p-q}$, which were phased in the haplotype segments.

$$\begin{aligned} V(e_{p+1}) &= \text{Max}_{e_p} V(e_p) P_t(e_{p+1}|e_p) P_e(g_{p+1}|e_{p+1}) \\ T(e_{p+1}) &= \text{argmax}_{e_p} V(e_p) P_t(e_{p+1}|e_p) P_e(g_{p+1}|e_{p+1}) \\ &\text{subject to} \\ T(e_{p+1})^q = T(T(...T(e_{p+1})...)) &= \ \hat{e}_{p+1-q} \text{ if } e_{p+1} \text{ is phased in } H_i^{\text{snp}} \end{aligned} \quad (17)$$

Supplementary Note 13: Multiway tree for enumeration of Eulerian paths.

The haplotype breakpoint graph is an undirected multigraph, for which we used the multiway tree to enumerate Eulerian paths by obtaining the combinations of pairing states of breakpoint edges[24]. Candidate genomes require that Eulerian paths alternate between SV/reference edges and segment edges (alternations between same edges or between SV and reference edges are not permitted) so that edge pairing is performed to follow the alternation criteria. From the root, we expand the multiway tree by processing the nodes of the haplotype graph in a breadth-first search manner (Supplementary Fig. 20). When branching the multiway tree, we measure information entropy to prioritise solutions with low entropies (having duplicated segments). Low-entropy paths indicate that a set of SVs is amplified at the same time through duplications, such as focal, chromosomal, or whole-genome duplications. Because we process nodes of the breakpoint graph individually, we consider the entropy in local breakpoints first, and the final entropy is measured on a chromosomal

level in the leaves. The enumeration of all Eulerian paths is an NP-hard problem, and it is not feasible for highly segmented samples. The entropy-based prioritisation enables the enumeration of Eulerian paths because we cut off branches with high entropies to obtain probable candidate genomes with minimum entropies.

Supplementary Note 14: Breakpoint graph construction for multi-sample data.

After constructing breakpoint graphs with the unified set, we classified SVs into private and shared SVs depending on the existence of raw SV evidence (discordant or split reads) (Supplementary Table 5). However, there are ambiguous cases in which raw SV evidence is not consistent with the breakpoint graph: 1) an SV edge is found only in a metastatic tumour, but only the primary tumour has discordant/split reads for the SV and 2) both tumours have raw SV evidence, but one of the tumours has no SV edge. In these cases, the SVs are considered as putative shared SVs that require fine-tuning of the breakpoint graphs. Therefore, we performed another round of iterative optimisation by adding putative shared SVs, and if the multiplicity is zero in one of the samples even after this round of iteration, we treat the SVs as false or unidentifiable and finally remove them. The steps of adding putative shared SVs and removing false/unidentifiable SVs also requires iterations until the graphs converge.

a

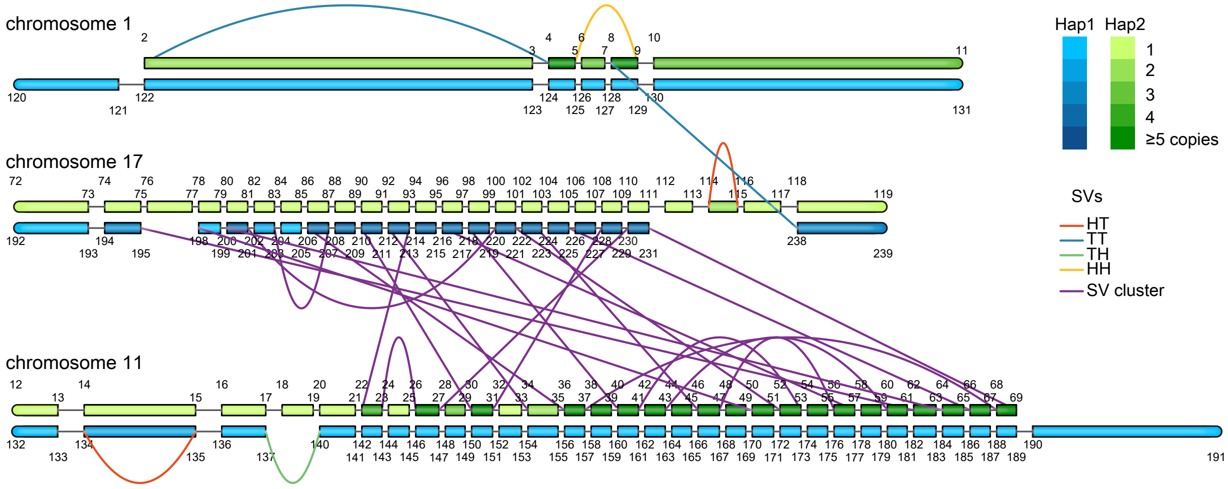

b

Index	Topology	Type	Mutiplicity	Path
Contig 1	CT+HSR/DM cluster	circular	9	230 231 69 68 67 66 65 64 63 62 61 60 59 58 57 56 55 54 53 52 51 50 49 48 47 46 45 44 43 42 41 40 39 38 37 36 206 207 208 209 210 211 212 213 214 215 216 217 218 219 220 221 222 223 224 225 226 227 228 229 230
Contig 2		circular	4	230 231 69 68 67 66 65 64 200 201 220 221 222 223 224 225 226 227 228 229 230
Contig 3		linear	1	225 224 223 222 221 220 219 218 40 41 42 43 60 61 62 63 64 65 66 67 68 69 231 230 229 228 227 226 225 224 223 222 221 220 219 218 217 216 57 56 47 46 45 44 43 42 41 40 39 38 37 36 206 207 208 209 210 211 212 213 214 215 216 217 218 219 220 221 222 223 224 225 226 227 228 229 230 231 69 68 67 66 65 64 63 62 61 60 43 42 41 40 218 219 220 221 222 223 224 225
Contig 4		linear	1	43 42 41 40 39 38 37 36 206 207 208 209 210 211 212 213 214 215 216 217 218 219 220 221 222 223 224 225 66 67 68 69 231 230 229 228 227 226 225 224 223 222 221 220 219 218 217 216 57 56 47 46 45 44 43 42 41 40 39 38 37 36 206 207 208 209 210 211 212 213 214 215 216 217 218 219 220 221 222 223 224 225 226 227 228 229 230 231 69 68 67 66 225 224 223 222 221 220 219 218 217 216 215 214 213 212 211 210 209 208 207 206 36 37 38 39 40 41 42 43
Contig 5		linear	1	220 221 52 53 54 55 56 57 58 59 60 61 62 63 64 65 66 67 68 69 231 230 229 228 227 226 225 224 223 222 221 220 219 218 217 216 215 214 213 212 34 35 36 37 38 39 40 41 53 52 51 50 198 199 200 201 202 203 204 205 206 207 208 209 210 211 212 213 214 215 216 217 218 219 220 221 222 223 224 225 226 227 228 229 230 231 69 68 67 66 65 64 63 62 195 194 193 192
Contig 6		linear	1	12 13 14 15 16 17 18 19 20 21 22 23 24 25 26 27 230 231 69 68 37 36 35 34 33 32 31 30 29 28 27 26 23 22 213 212
Contig 7		linear	1	72 73 74 75 76 77 78 79 80 81 82 83 84 85 86 87 88 89 90 91 92 93 94 95 96 97 98 99 100 101 102 103 104 105 106 107 108 109 110 111 112 113 114 115 114 115 116 117 118 119
Contig 8		linear	1	132 133 134 135 134 135 136 137 140 141 142 143 144 145 146 147 148 149 150 151 152 153 154 155 156 157 158 159 160 161 162 163 164 165 166 167 168 169 170 171 172 173 174 175 176 177 178 179 180 181 182 183 184 185 186 187 188 189 190 191
Contig 9		linear	1	11 10 9 8 7 6 5 4 3 2 4 5 9 8 238 239
Contig 10		linear	1	120 121 122 123 124 125 126 127 128 129 130 131

Supplementary Fig. 1: An example of the rearrangement topologies and derived cancer genome karyotypes. **a,** The haplotype graph of chromosomes 1, 11, and 17 of the TCGA-A1-A0SM sample. InfoGenomeR found an SV cluster (purple) from the TCGA-A1-A0SM sample and classified it into the CT+HSR/DM cluster. **b,** The karyotype paths derived from the haplotype graph. The paths were denoted by a series of node numbers of the haplotype graph. Six paths were derived from the CT+HSR/DM cluster, and contig 1 and contig 2 were circular types involved with the DM component.

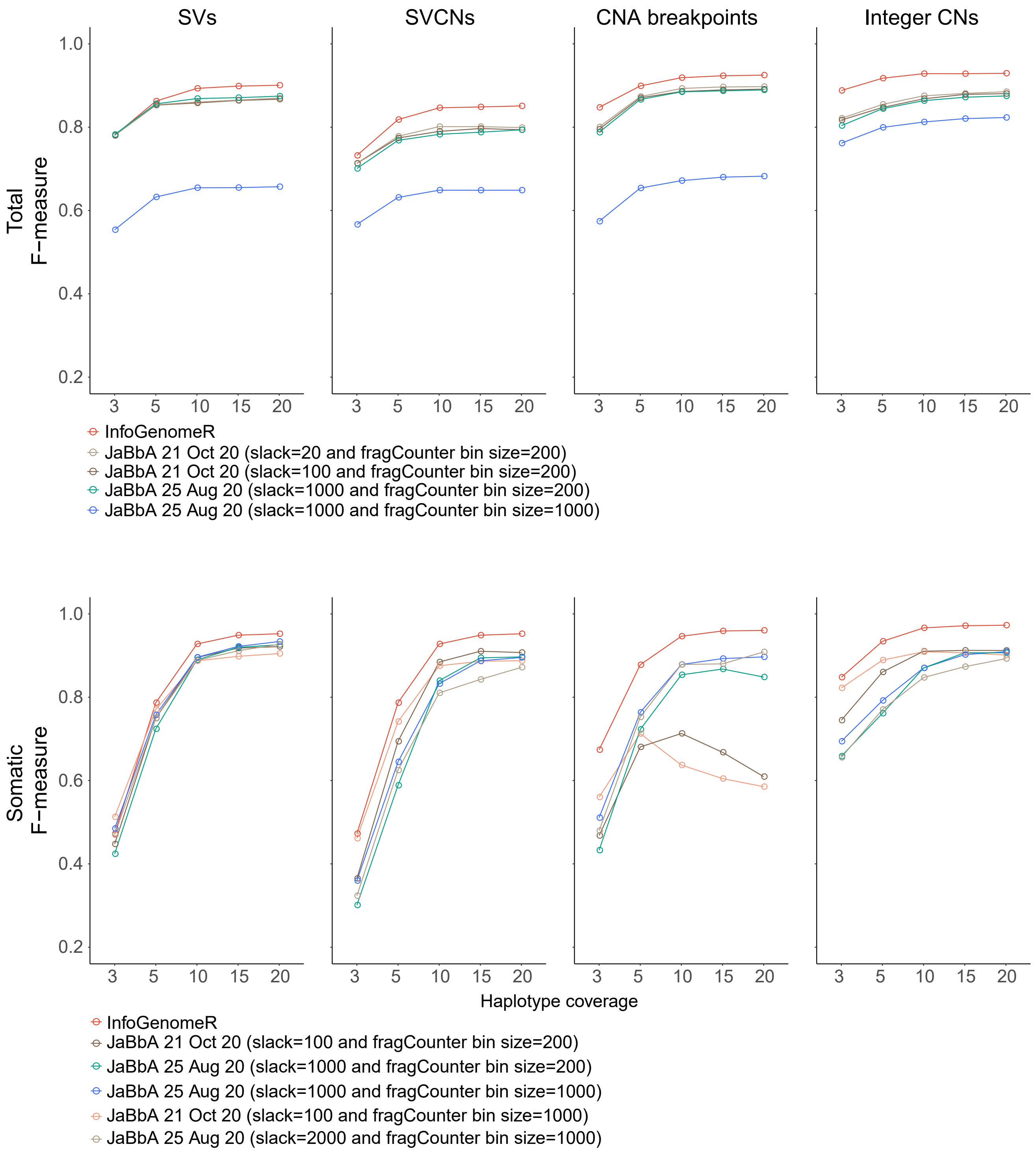

Supplementary Fig. 2: JaBbA performance with simulated data sets depending on hyperparameters. F-measures for SVs, SVCNs, CNA breakpoints, and integer CNs were measured using various JaBbA hyperparameter settings. Two different JaBbA codes (downloaded on 21 October 20 and 25 August 20) were used with the recommended settings. The X-axis denotes the haplotype coverage (3X to 20X), representing the mean number of reads aligned to a nucleotide in a haplotype.

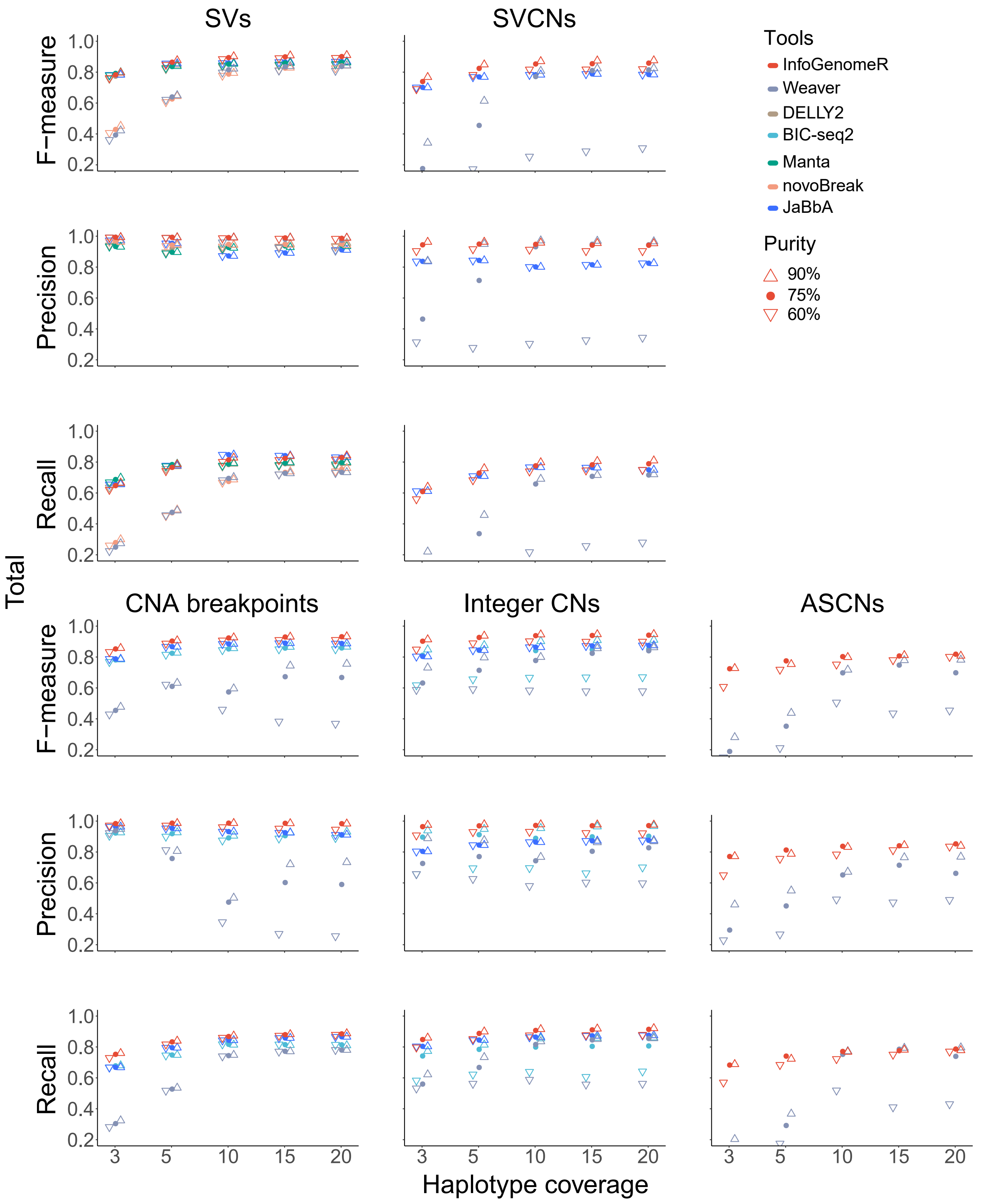
SVs
SVCNs
Tools
InfoGenomeR
Weaver
DELLY2
BIC-seq2
Manta
novoBreak
JaBbA
Purity
90%
75%
60%
F−measure
Precision
Recall
Total
CNA breakpoints
Integer CNs
ASCNs
Haplotype coverage
3
5
10
15
20
0.2
0.4
0.6
0.8
1.0

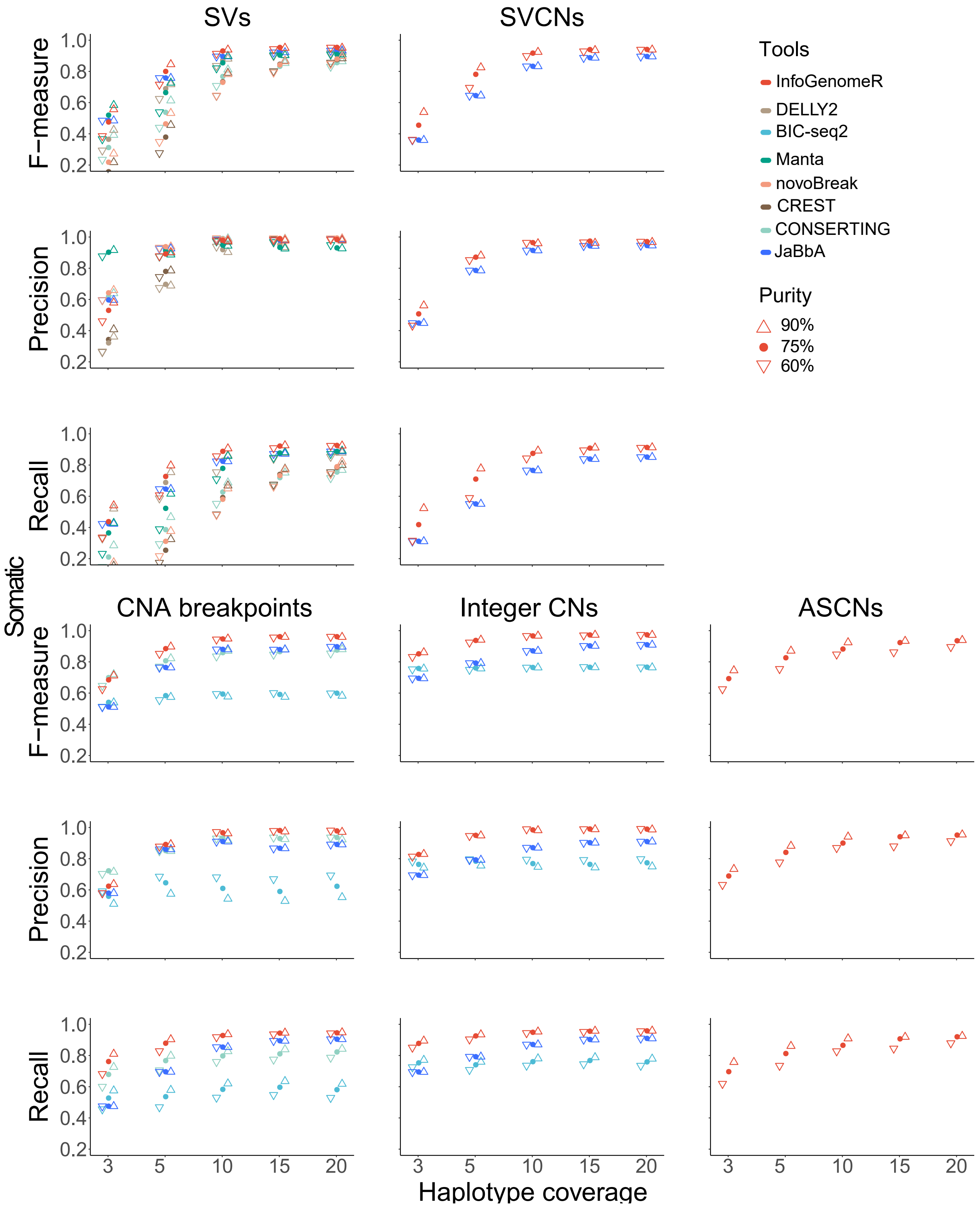

Supplementary Fig. 3: Performance comparison depending on cancer purities. Variant detection tools exhibit varying performances depending on the tumour purity with which cancer genomes were simulated; 0.6 (down-pointing triangle), 0.75 (circle), and 0.9 (up-pointing triangle).

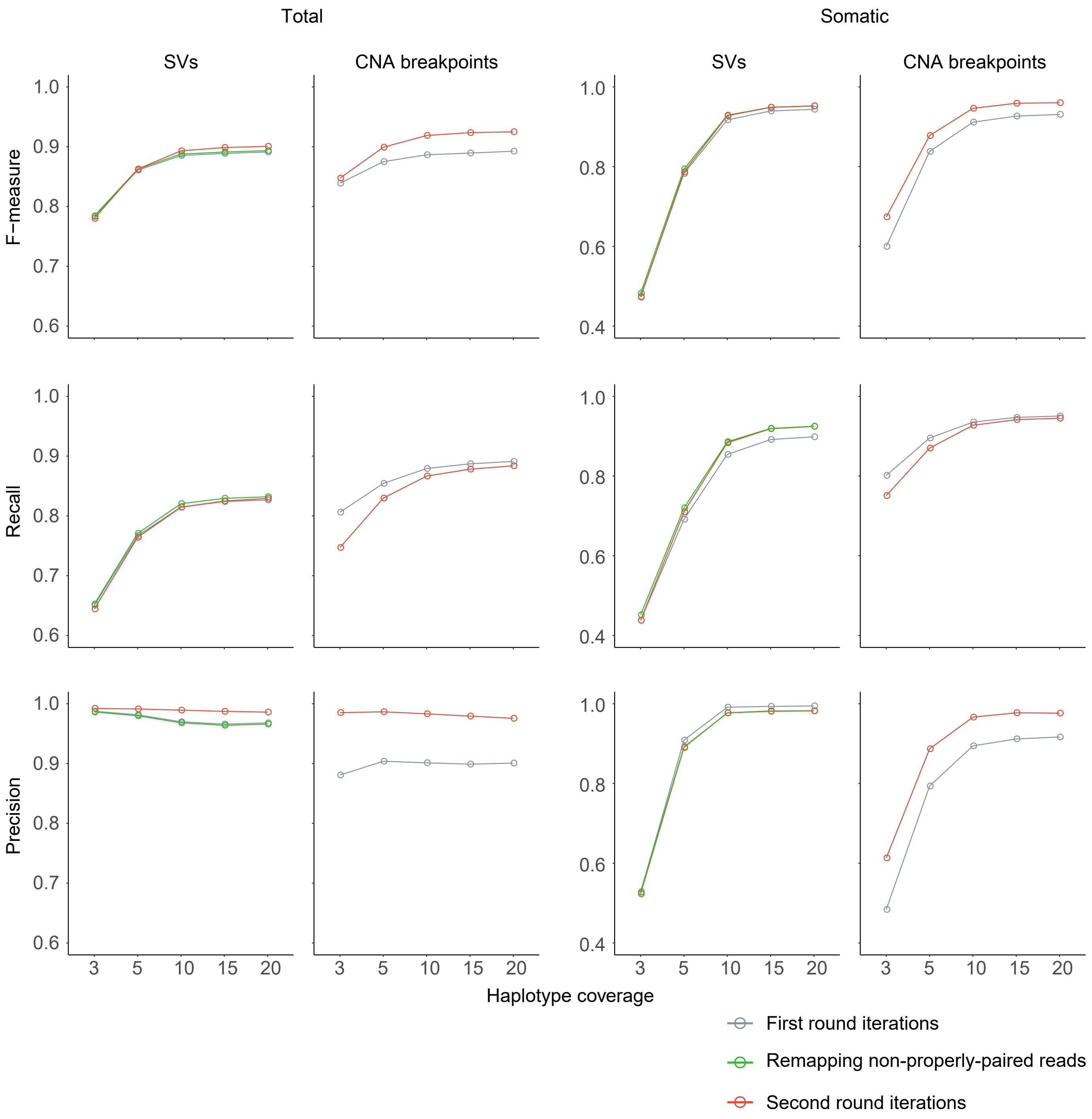

Supplementary Fig. 4: SV and CNA calling performance during InfoGenomeR iterations. F-measures were compared among the three time points: the end of the first round iterations (grey), the intermediate step of remapping nonproperly paired reads (green), and the end of second round iterations (red).

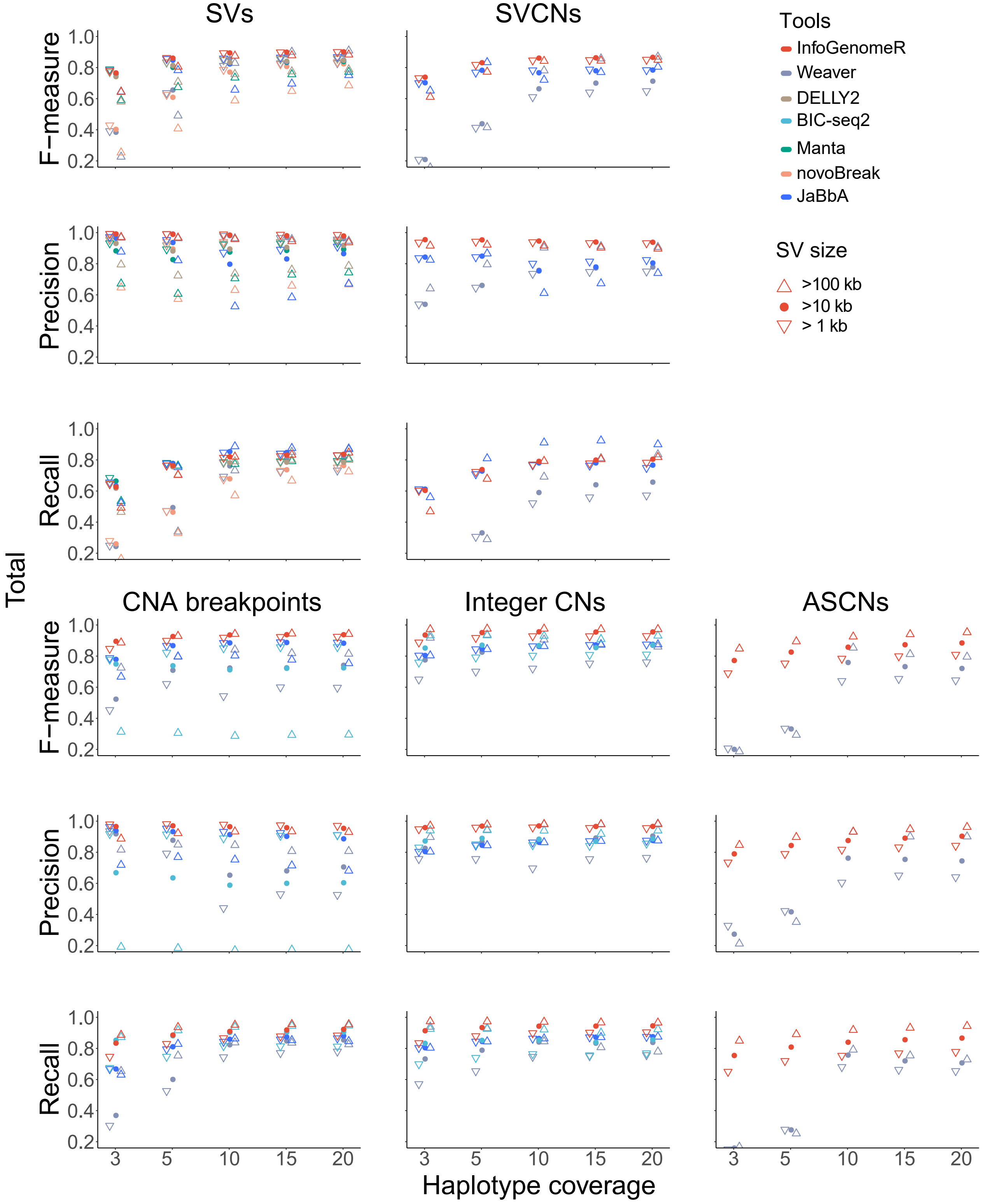
SVs
SVCNs
Tools
InfoGenomeR
Weaver
DELLY2
BIC-seq2
Manta
novoBreak
JaBbA
SV size
>100 kb
>10 kb
> 1 kb
F−measure
Precision
Recall
Total
CNA breakpoints
Integer CNs
ASCNs
Haplotype coverage
3
5
10
15
20
0.2
0.4
0.6
0.8
1.0

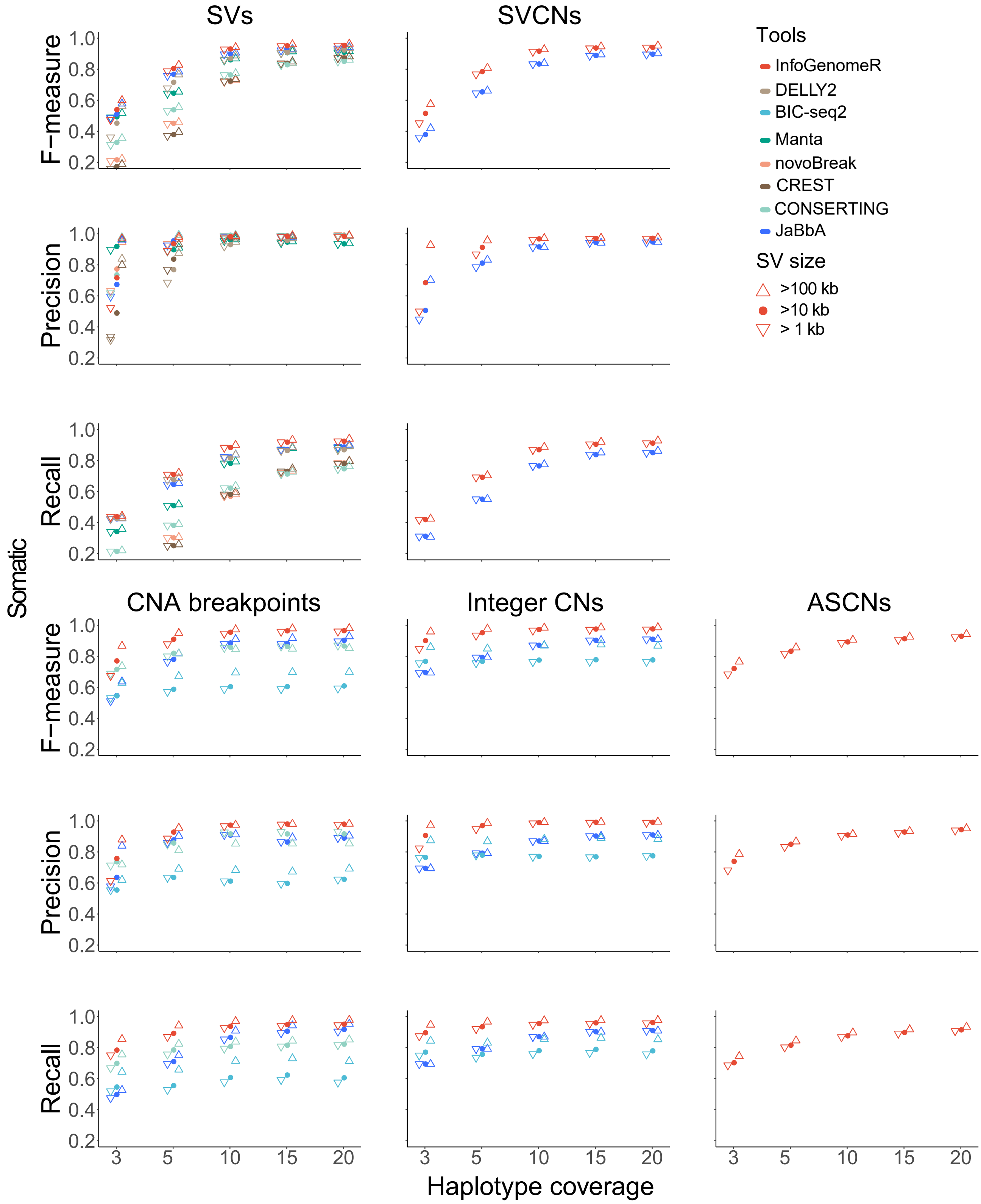

Supplementary Fig. 5: Performance comparison depending on variant sizes. Variant detection tools exhibit varying performances depending on the minimum variant size: ≥1kb (down-pointing triangle), ≥10kb (circle), and ≥100kb (up-pointing triangle).

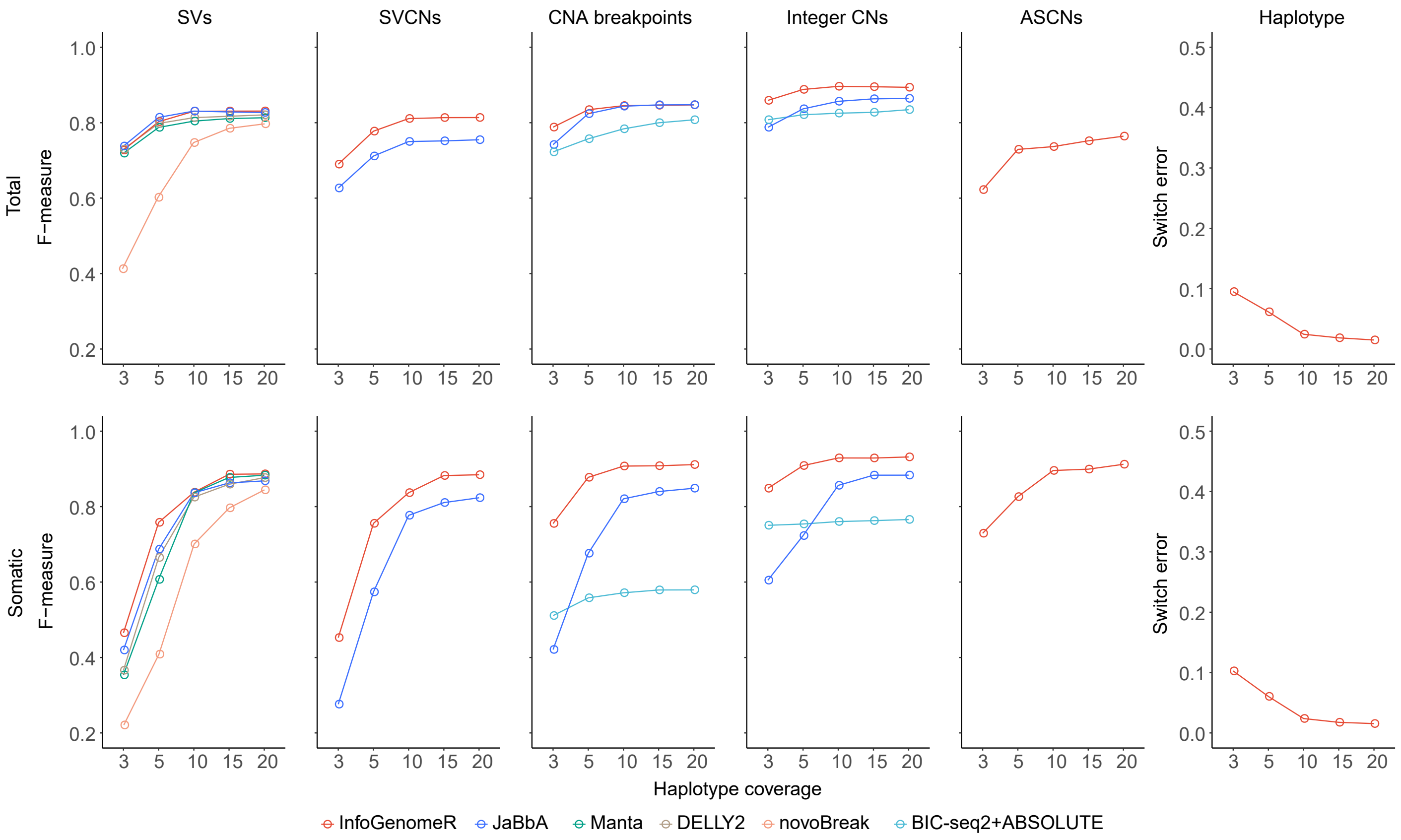

Supplementary Fig. 6: InfoGenomeR performance with GRCh38 simulated data sets. F-measures were compared among the various variant-calling tools for five variant-calling categories (SVs, SVCNs, CNA breakpoints, integer CNs, and ASCNs) and switch error rates for haplotype, with controls (somatic variants), and without controls (total variants including germline and somatic variants). The X-axis denotes the haplotype coverage (3X to 20X), representing the mean number of reads aligned to a nucleotide in a haplotype.

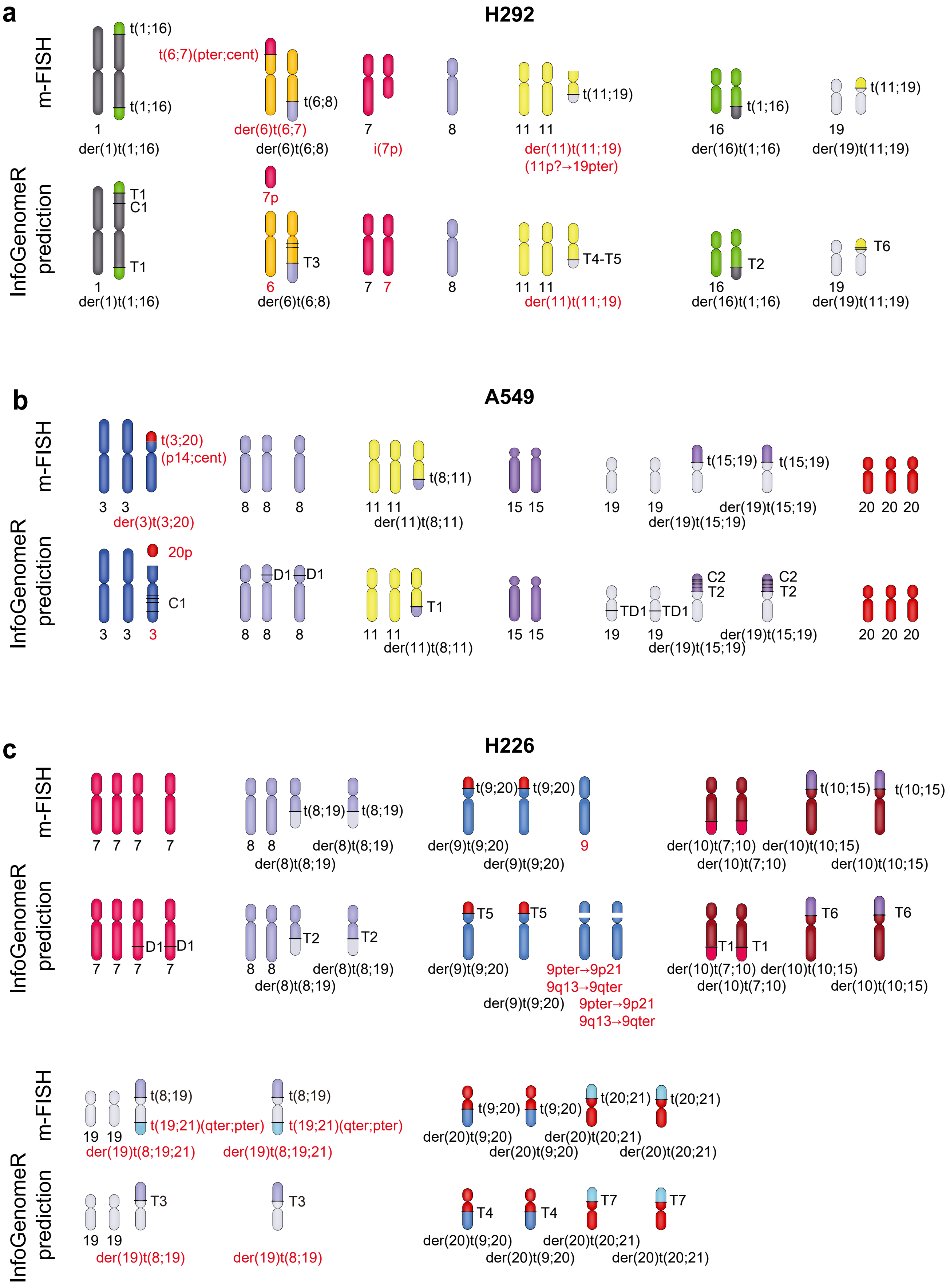
a
H292
m-FISH
InfoGenomeR prediction
t(1;16)
t(1;16)
t(6;7)(pter;cent)
t(6;8)
t(11;19)
t(1;16)
t(11;19)
1
der(1)t(1;16)
der(6)t(6;7)
der(6)t(6;8)
7
i(7p)
8
11 11
der(11)t(11;19)
(11p?→19pter)
16
der(16)t(1;16)
19
der(19)t(11;19)
T1
C1
T1
7p
T3
T4-T5
T2
T6
1
der(1)t(1;16)
6
der(6)t(6;8)
7 7
8
11 11
der(11)t(11;19)
16
der(16)t(1;16)
19
der(19)t(11;19)
b
A549
m-FISH
InfoGenomeR prediction
t(3;20)
(p14;cent)
t(8;11)
t(15;19)
t(15;19)
3 3
der(3)t(3;20)
8 8 8
11 11
der(11)t(8;11)
15 15
19
19
der(19)t(15;19)
der(19)t(15;19)
20 20 20
20p
C1
D1
D1
T1
C2
T2
C2
T2
TD1
TD1
3 3 3
8 8 8
11 11
der(11)t(8;11)
15 15
19
19
der(19)t(15;19)
der(19)t(15;19)
20 20 20
c
H226
m-FISH
InfoGenomeR prediction
7 7 7 7
t(8;19)
t(8;19)
8 8
der(8)t(8;19)
der(8)t(8;19)
t(9;20)
t(9;20)
der(9)t(9;20)
der(9)t(9;20)
9
t(10;15)
t(10;15)
der(10)t(7;10)
der(10)t(10;15)
der(10)t(7;10)
der(10)t(10;15)
D1
D1
7 7 7 7
T2
T2
8 8
der(8)t(8;19)
der(8)t(8;19)
T5
T5
der(9)t(9;20)
der(9)t(9;20)
9pter→9p21
9q13→9qter
9pter→9p21
9q13→9qter
T1
T1
T6
T6
der(10)t(7;10)
der(10)t(10;15)
der(10)t(7;10)
der(10)t(10;15)
m-FISH
InfoGenomeR prediction
t(8;19)
t(19;21)(qter;pter)
t(8;19)
t(19;21)(qter;pter)
19 19
der(19)t(8;19;21)
der(19)t(8;19;21)
t(9;20)
t(9;20)
t(20;21)
t(20;21)
der(20)t(9;20)
der(20)t(9;20)
der(20)t(20;21)
der(20)t(20;21)
T3
T3
19 19
der(19)t(8;19)
der(19)t(8;19)
T4
T4
T7
T7
der(20)t(9;20)
der(20)t(9;20)
der(20)t(20;21)
der(20)t(20;21)

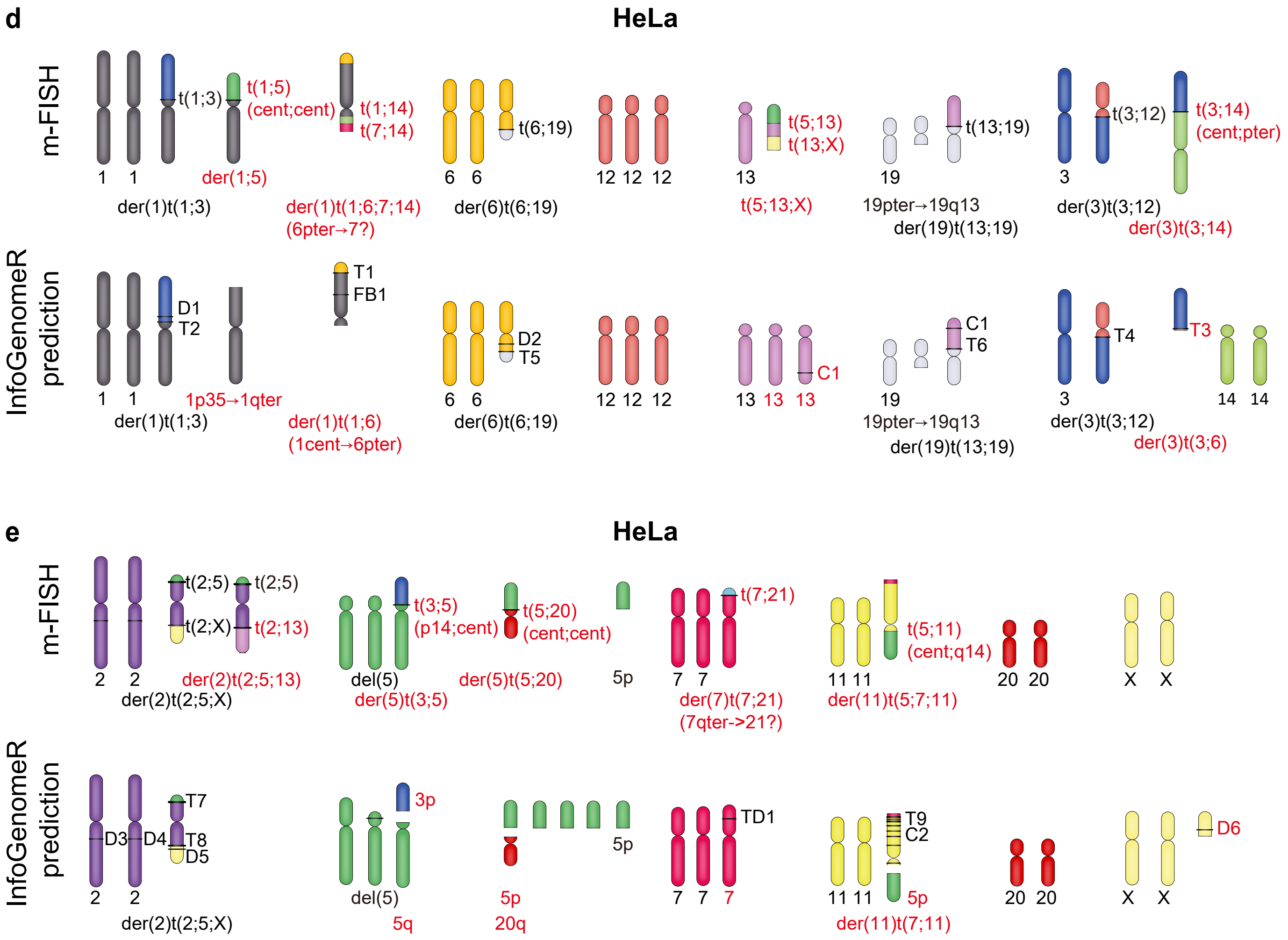

Supplementary Fig. 7: Comparison between multiplex fluorescence in situ hybridisation (m-FISH) karyotypes and reconstructed karyotypes of cancer cell lines. The reconstructed karyotypes (bottom) are compared with m-FISH karyotypes (top). **a-e,** The karyotypes of the H292 (a), A549 (b), H226 (c), HeLa (d-e) cell lines are shown. Differences between true and reconstructed karyotypes are annotated with karyotypic notations (Supplementary Table 3) (red). SVs included in karyotype analysis are denoted by D (deletion), TD (tandem duplication), T (translocation), FB (fold-back inversion), and C (complex SVs). The detailed haplotype-graph annotations of SVs are shown in Figure 3 in the main manuscript.

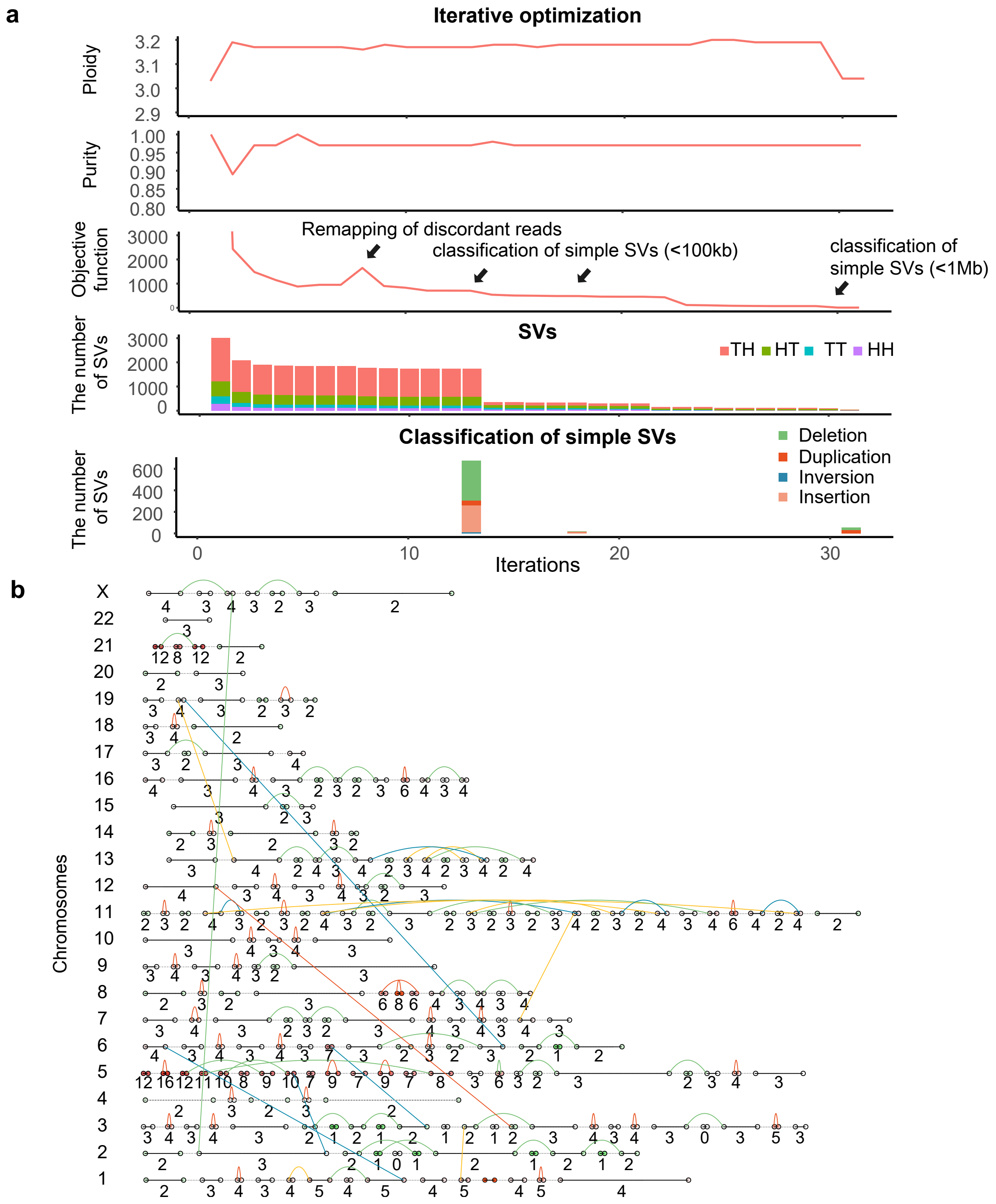

Supplementary Fig. 8: Breakpoint construction of the HeLa genome. a, The iterative optimisation for the breakpoint graph construction. The objective function is reduced by removing false positive SVs, and the cancer purity and ploidy are adjusted during the iterations. The step for adding putative true SVs increases the objective function temporarily but reduces the objective function further during the later iterations. Short simple SVs (<100kb) were removed to simplify the breakpoint graph. **b,** The breakpoint graph of the HeLa genome. The circles represent the head and tail nodes, and the black lines and coloured lines represent the segment edges and SV edges, respectively. The reference edges are represented by dotted lines between nodes. The numbers under the segment edges represent integer CNs.

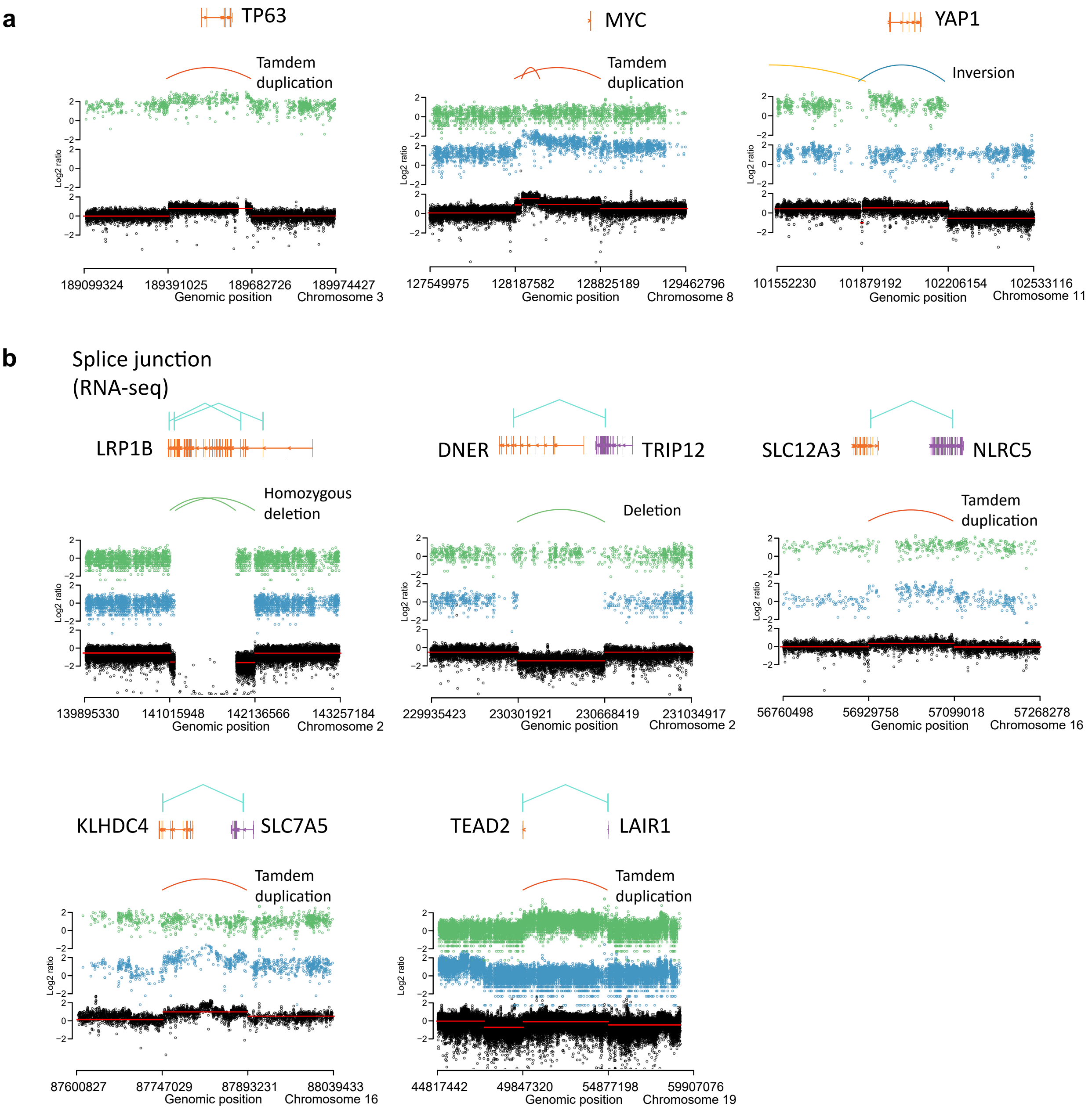

Supplementary Fig. 9: Splice junction analysis of the HeLa transcriptome. a, CNAs of cervical cancer-related genes, and **b,** splice junctions between exons discovered within an RNA-seq dataset. SVs were consistently found with total (black) and haplotype-specific CNAs (blue and green). Five SVs occurred between exons (LRP1B, DNER-TRIP12, SLC12A3-NLRC5, KLHDC4-SLC7A5, and TEAD2-LAIR1), among which novel splice junctions were validated using RNA-seq (cyan).

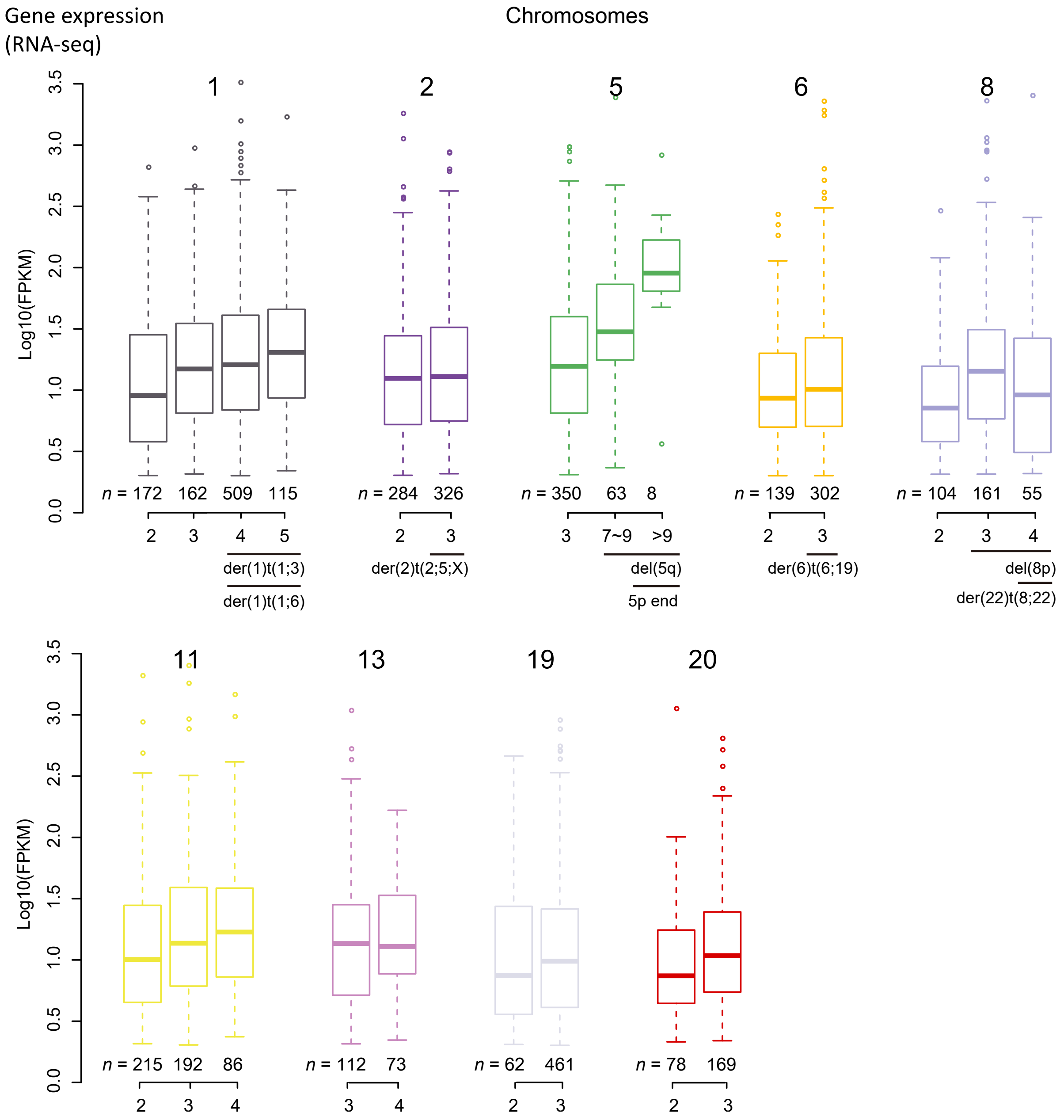

Supplementary Fig. 10: Gene expression analysis of the HeLa transcriptome. Gene expression of derivative chromosomes. The X-axis represents copy numbers of genes proportional to the additional copies from the derivative chromosomes. The expression levels of genes with >2 FPKM values were examined across chromosomes. The number of genes is denoted under each box plot. The boxplot centre lines are medians, box limits are upper and lower quantiles, whiskers are 1.5x interquantile ranges, and dots are outliers.

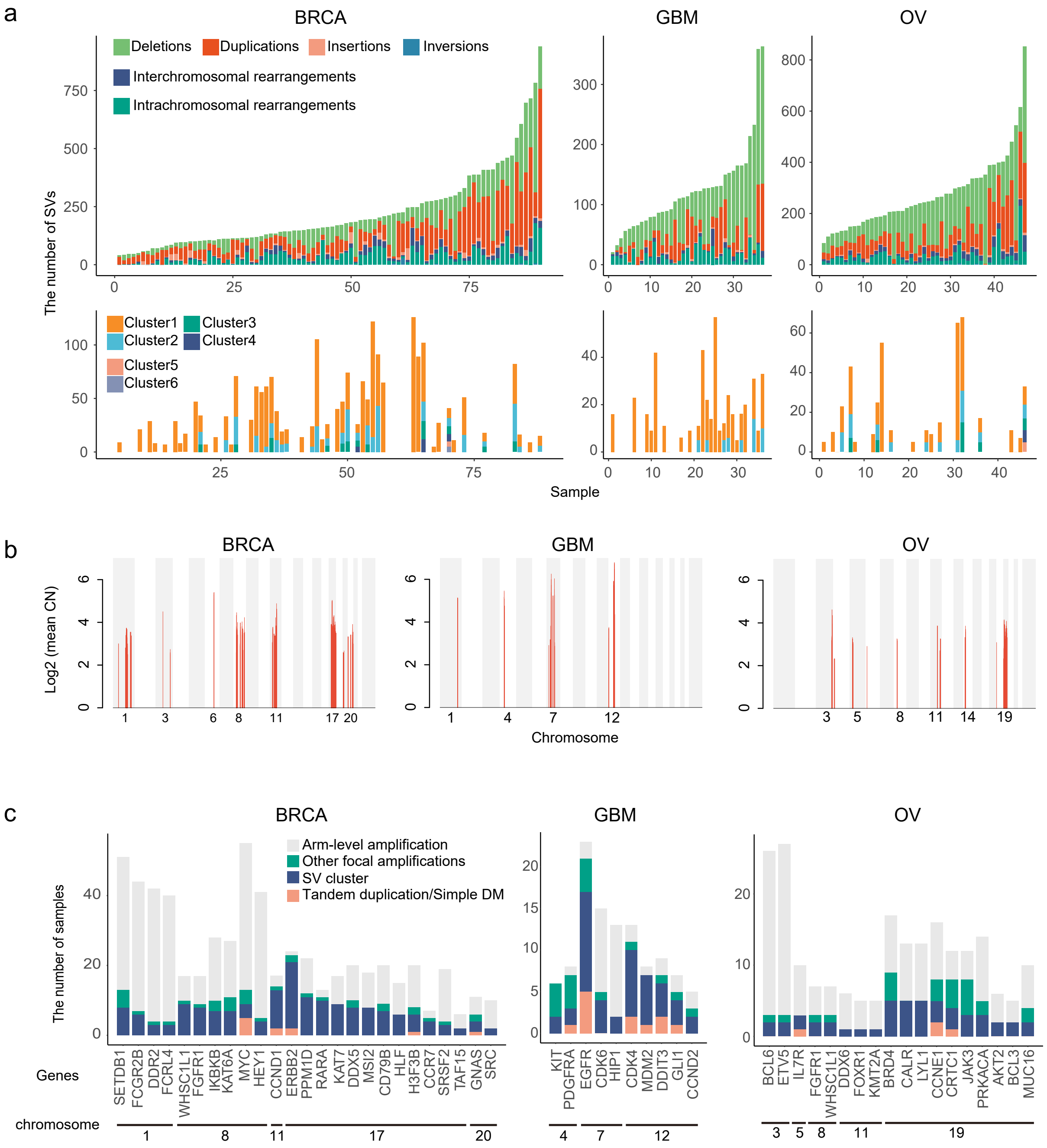

Supplementary Fig. 11: Landscape of somatic SVs and SV clusters. a, Somatic SVs in BRCAs, GBMs, and OVs. Simple SVs (deletions, duplications, insertions, and inversions) and complex SVs (interchromosomal and intrachromosomal rearrangements) are shown for each sample. SV clusters from the clustering analysis of complex SVs are shown below. **b,** Amplification peaks in SV clusters. The mean copy number of each gene amplified in SV clusters was measured ($\log_2$ scale). **c,** Oncogenes amplified in SV clusters. Genes are listed in order of the number of amplification events by SV clusters (blue) for each chromosome. Other focal amplifications and tandem duplications/simple double minutes are shown in green and apricot, respectively.

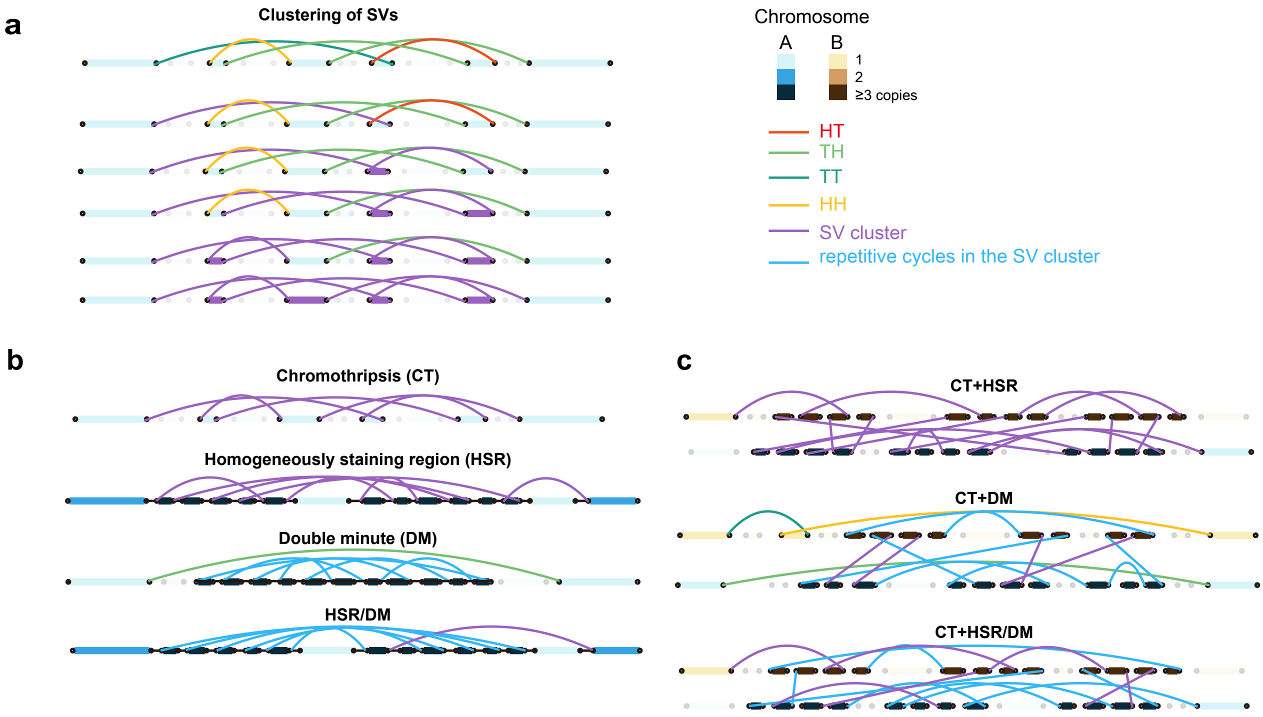

Supplementary Fig. 12: Classification of SV clusters. **a,** Clustering of complex SVs in the breakpoint graph. Complex SVs that can form a derivative path are clustered together, and the SV cluster can be found using a breadth-first search. The path requires alternations between the segment edges and reference/SV edges. **b,** Four basic classes of SV clusters: one deletion type, chromothripsis (CT), and three amplification types (HSR, DM, and HSR/DM). **c,** Combinations of the deletion and amplification types. Multiple chromosomes can be involved in the same SV cluster, resulting in a complex derivative chromosome.

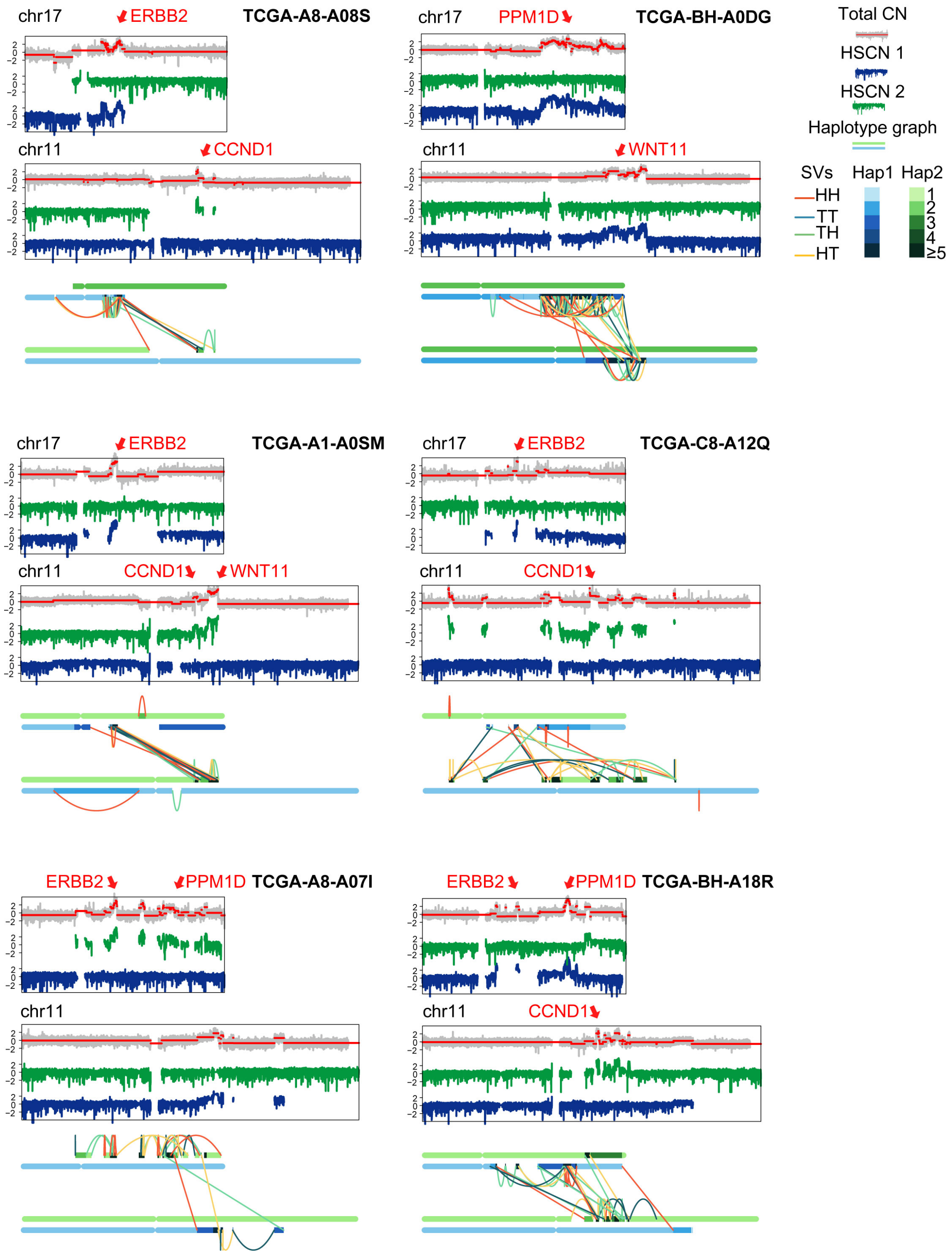
chr17
ERBB2
TCGA-A8-A08S
chr11
CCND1
chr17
PPM1D
TCGA-BH-A0DG
chr11
WNT11
Total CN
HSCN 1
HSCN 2
Haplotype graph
SVs
Hap1
Hap2
HH
TT
TH
HT
1
2
3
4
≥5
chr17
ERBB2
TCGA-A1-A0SM
chr11
CCND1
WNT11
chr17
ERBB2
TCGA-C8-A12Q
chr11
CCND1
ERBB2
PPM1D
TCGA-A8-A07I
chr11
ERBB2
PPM1D
TCGA-BH-A18R
chr11
CCND1

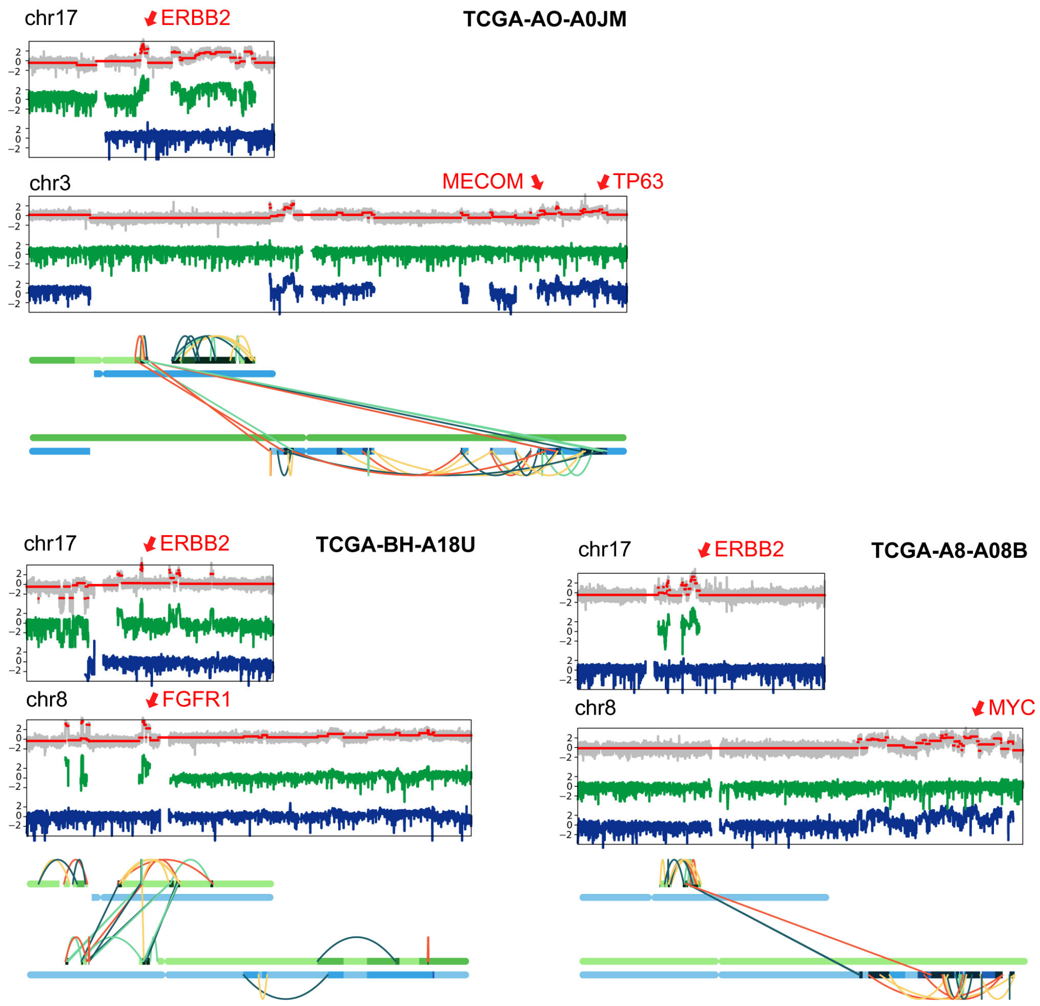

Supplementary Fig. 13: Haplotype graphs with SV clusters of BRCAs. Haplotype graphs are shown together with total CNAs (grey) and haplotype-specific CNAs (blue and green) on the log2 scale and the oncogenes in amplified focal segments are annotated (red arrow).

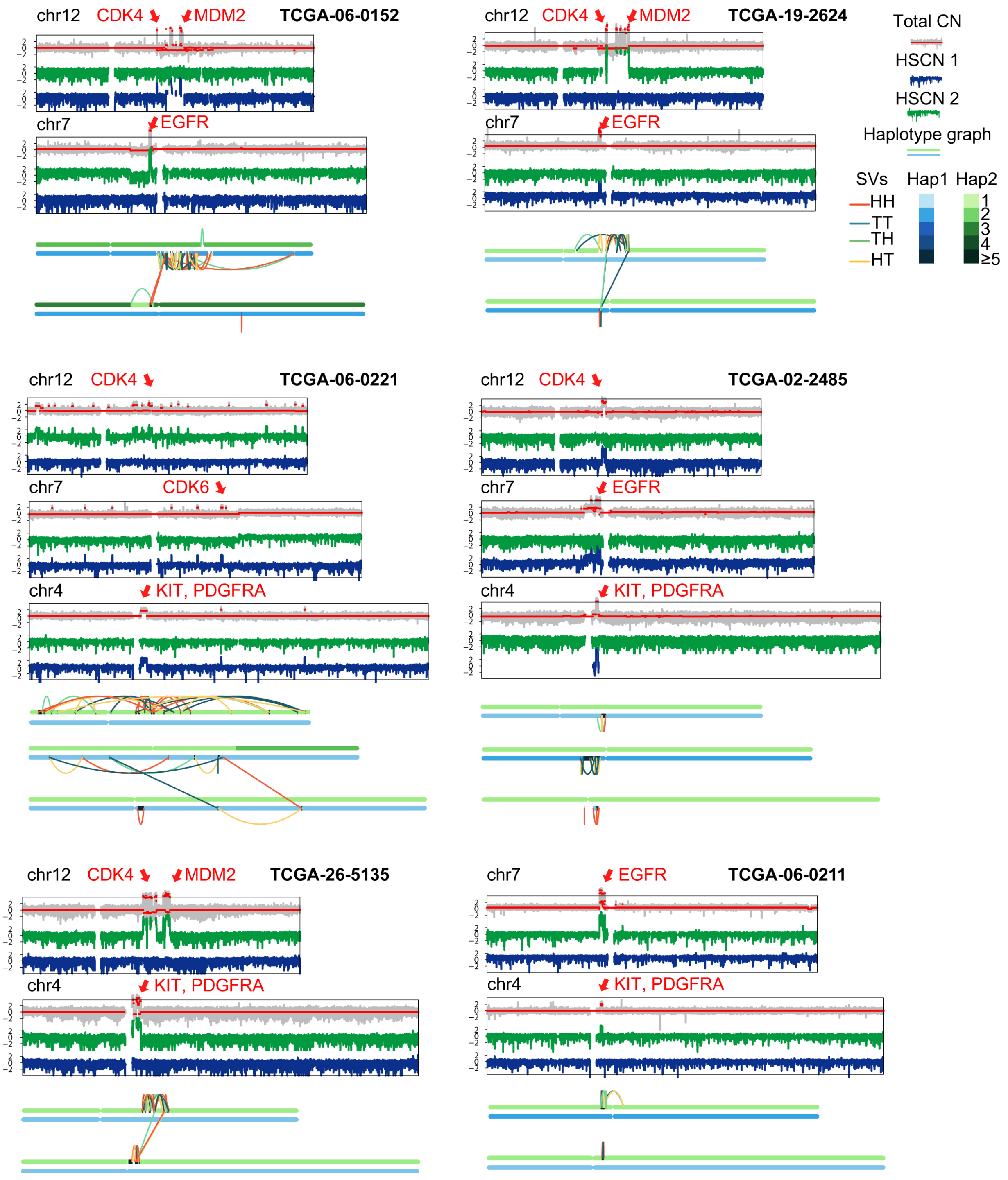

Supplementary Fig. 14: Haplotype graphs with SV clusters of GBMs. Haplotype graphs are shown together with total CNAs (grey) and haplotype-specific CNAs (blue and green) on the log2 scale and the oncogenes in amplified focal segments are annotated (red arrow).

Chromosome 19 (OV)

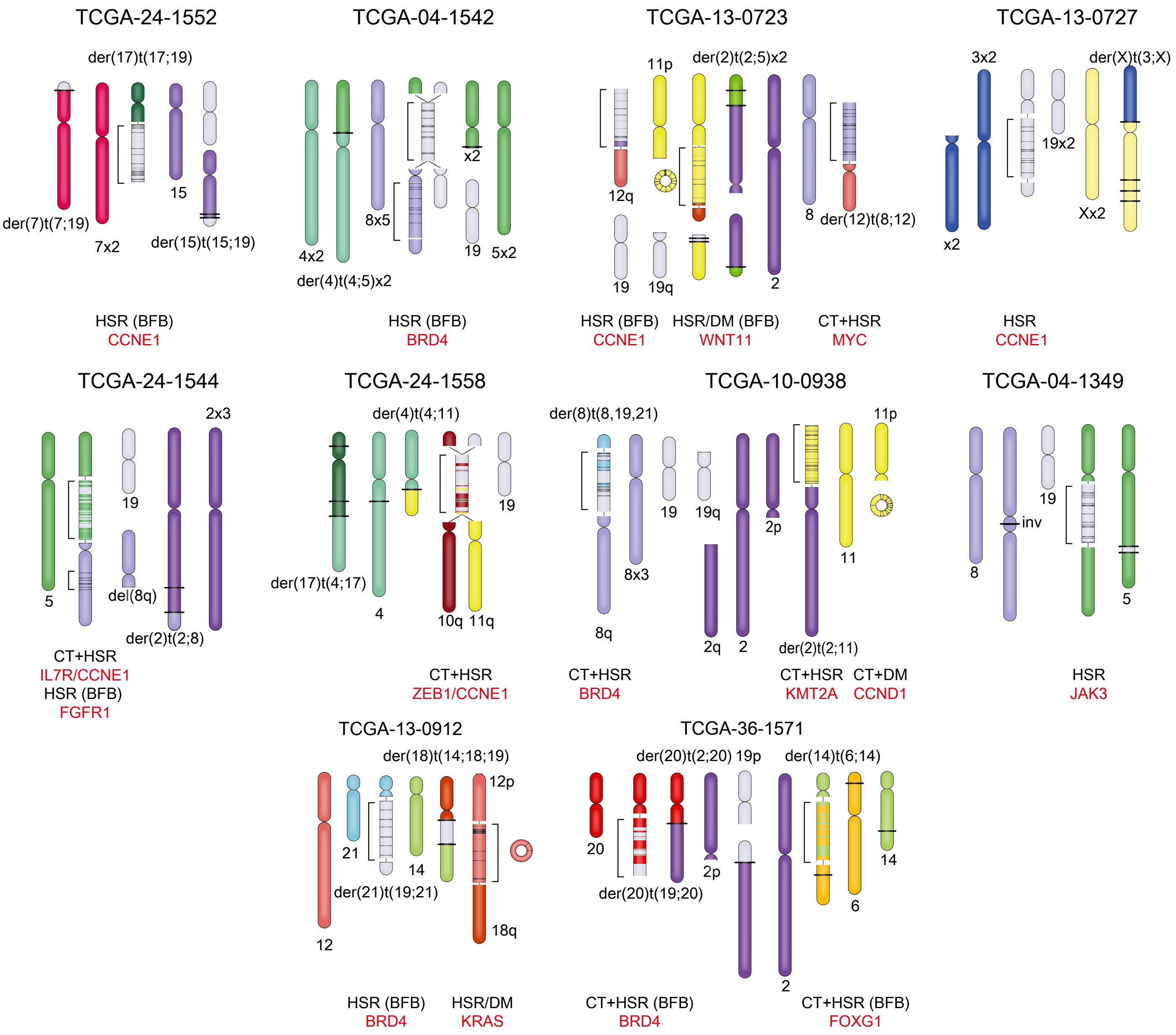

Supplementary Fig. 15: The karyotypic landscape of OVs. Karyotypic scenarios of OVs and SV clusters are shown, and SV clusters enriched with fold-back inversions are annotated using a breakage-fusion-bridge (BFB) cycle. BFB cycles induce inverted repeats elongating the break ends and terminate by receiving a telomere or a chromosomal arm from other chromosomes. BFBs and CTs can be observed together (TCGA-24-1544 and TCGA-36-1571) generating derivative chromosomes with interchromosomal HSRs.

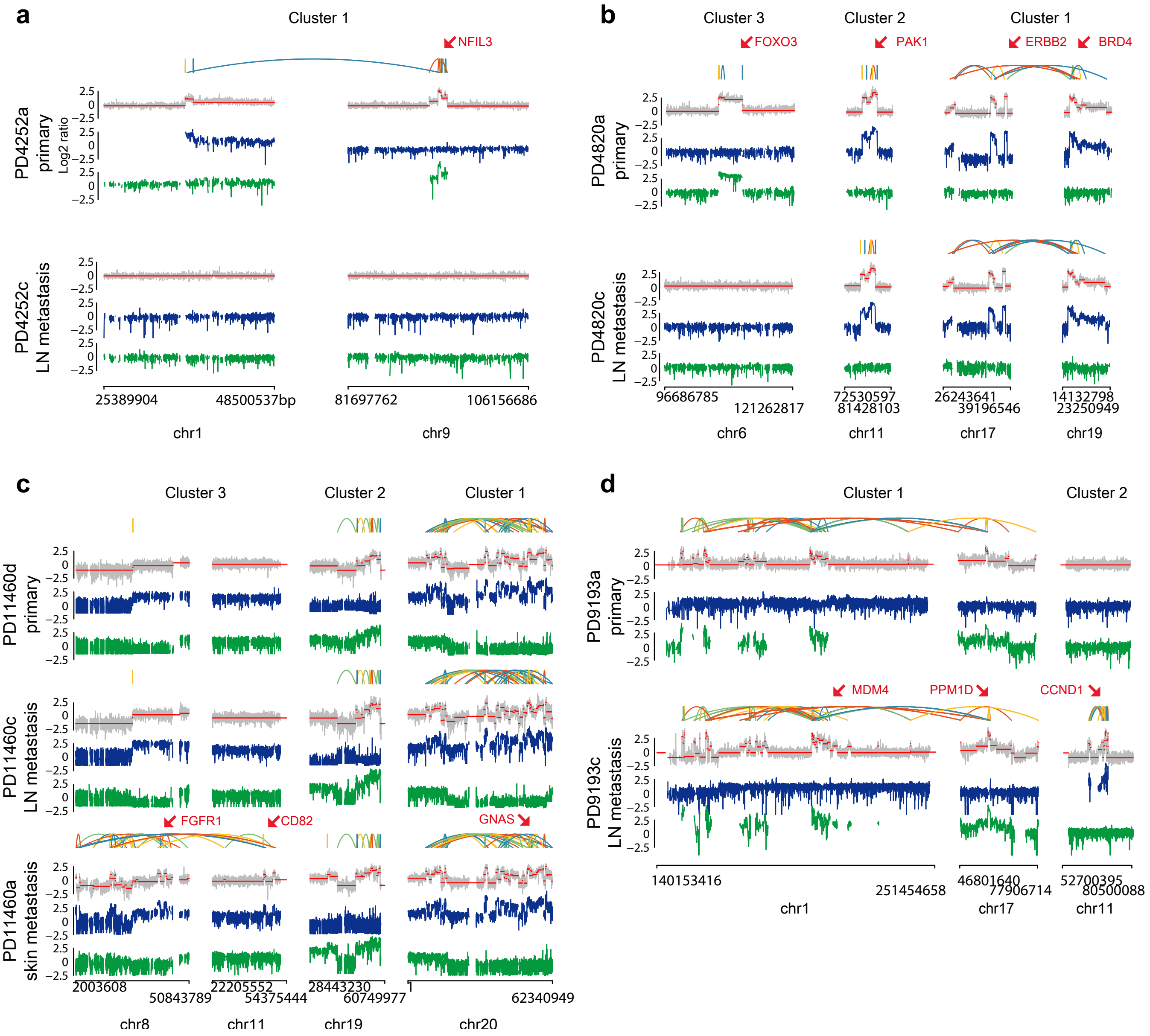

Supplementary Fig. 16: The evolution of SV clusters in breast cancers and metastases. SV clusters (coloured lines), total CNAs (grey), and haplotype-specific CNAs (blue and green) change during tumour evolution. **a,** A primary evolution of cluster 1 (PD4252a). Cluster 1 is accompanied by LOH deletions in chromosomes 1 and 9. Focal amplification of NFIL3 was generated in the primary tumour. **b,** A primary evolution of cluster 3 (PD4820a). The primary tumour and LN metastasis have the same SV clusters (cluster 1 and cluster 2), and cluster 3 is generated in the primary tumour in chromosome 6. **c,** A metastatic evolution of cluster 3 (PD11460a. Multiple focal amplifications encompassing FGFR1 and CD82 are generated during skin metastasis. **d,** A metastatic evolution of cluster 2 (PD9193c. Cluster 1 in the primary tumour (PD9193a) is inherited intact to the LN metastasis, and focal amplifications encompassing CCND1 are generated in the LN metastasis with LOH deletions.

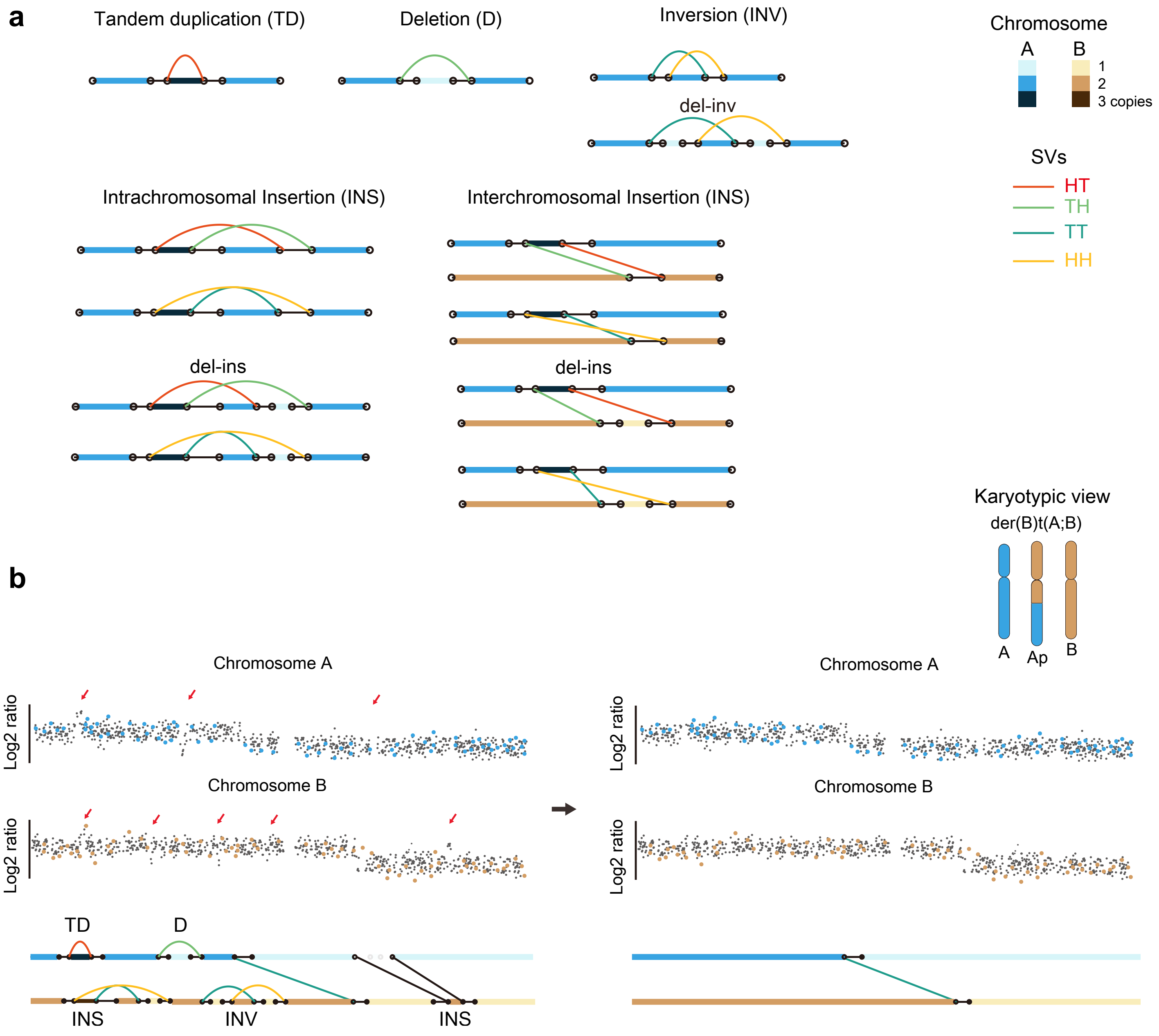

Supplementary Fig. 17: Classification of simple SVs and simplification of the breakpoint graph. a, The breakpoint graph showing simple SVs (tandem duplications, deletions, inversions, and insertions). For inversions and insertions, two SV edges were classified into a single event. **b,** The simplification process removed small simple SVs (<100 kb) from the breakpoint graph. The upper plots show the read depths of total copy number bins (black dots) and heterozygous SNPs (coloured dots). The depth information of heterozygous SNPs is sparse and can be absent within the segment of simple SVs. The simplification process removes the copy number bins of all the small simple SVs (red arrows) and reconstructs the breakpoint graph without them.

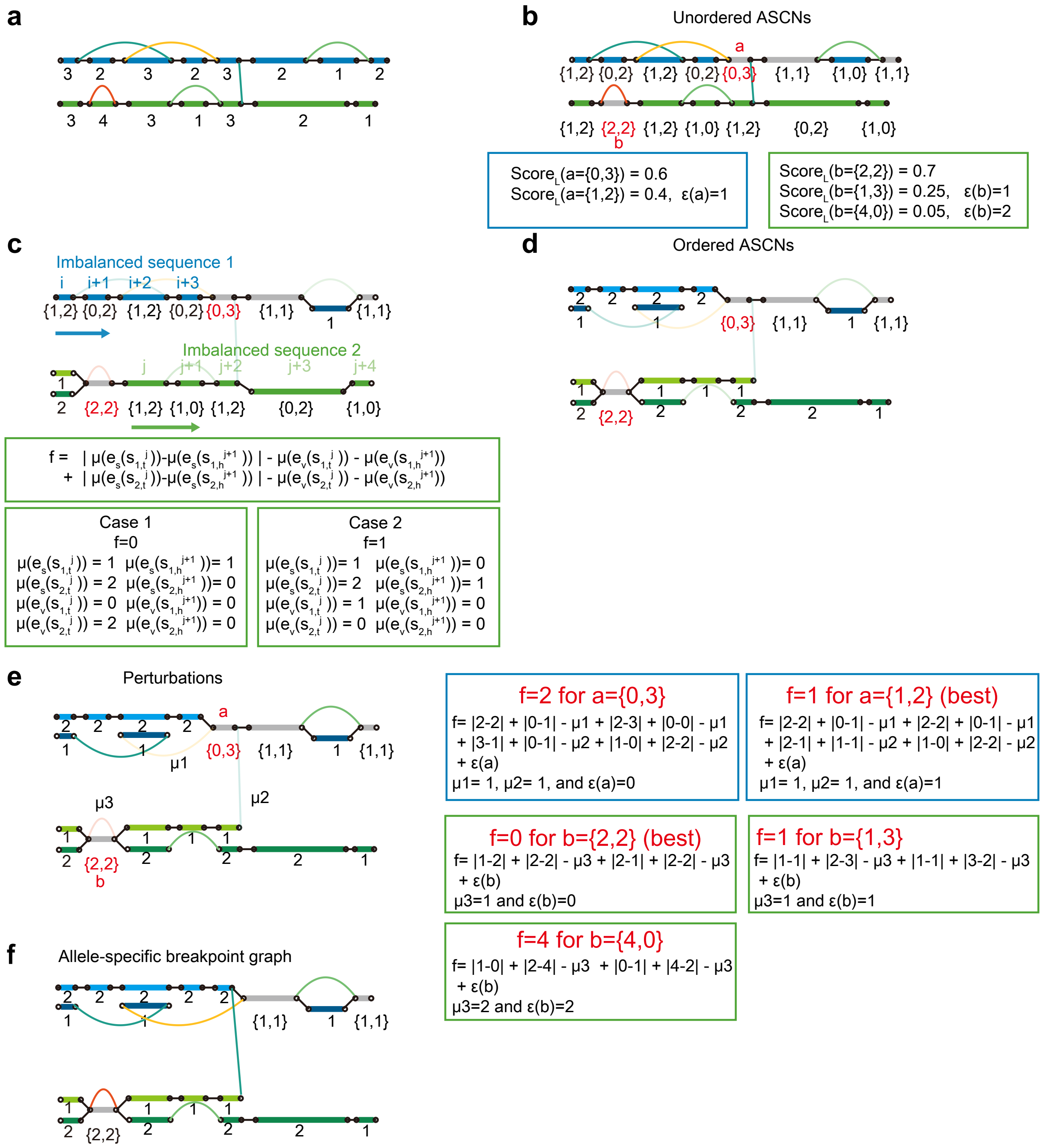

Supplementary Fig. 18: Construction of the allele-specific graph. **a,** The breakpoint graph constructed from total copy number information. **b,** Measurements of ASCNs and low-confidence segments(red). For the low-confidence segments with a maximum likelihood score of <0.8, the other possible ASCNs are searched with a penalty, ε. **c,** Optimisation with allelic switches in the imbalanced sequences (i, i+1, ..., i+3, and j, j+1, ..., j+4). A snapshot of the optimisation process between the jth and j+1th segments is shown. **c,** The ordered states of the imbalanced AS segments after optimisation of the imbalanced AS segments. **e,** Optimisation using penalty terms according to the likelihood scores of low-confidence ASCNs. **f,** The final allele-specific graph constructed from the allele-specific copy number information.

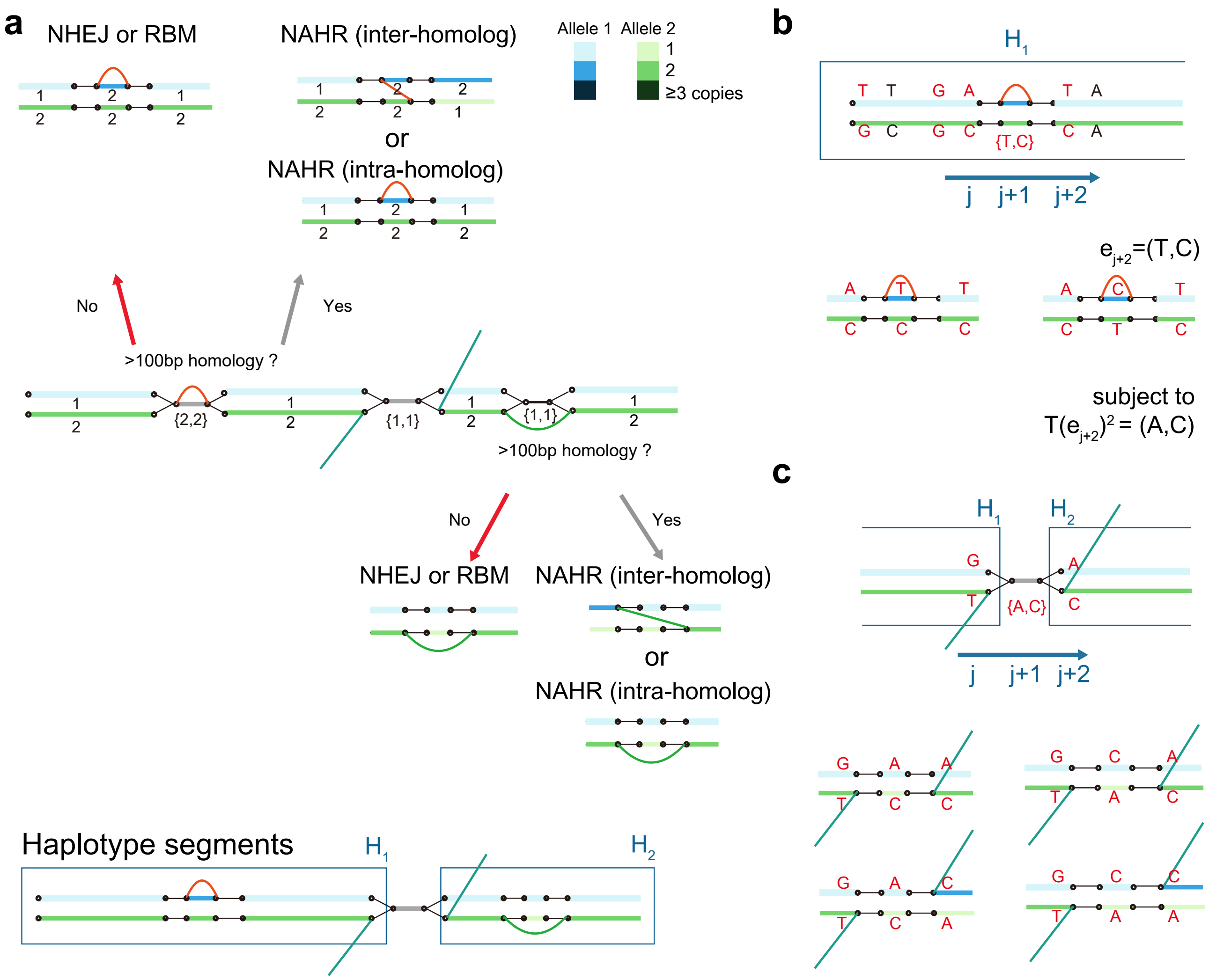

Supplementary Fig. 19: Haplotype segments and haplotype phasing. a, Classification of nonhomologous SVs and haplotype segments defined using allelic imbalances and nonhomologous SVs. Here, tandem duplication and deletion (<100 bp homology) are assumed to occur in an allele through nonhomologous mechanisms, such as NHEJ and RBM, and two haplotype segments, H_1 and H_2 are defined from them. **b,** Haplotype phasing in the same haplotype segment. Heterozygous SNPs in the same haplotype segment are phased through allelic imbalance (here, e_j and e_{j+2}), and the Viterbi algorithm is constrained to follow the previous phased state. **c,**Haplotype phasing between haplotype segments. Here, there are four possible phased states, and the Viterbi algorithm finds the most likely haplotype based on the population haplotypes.

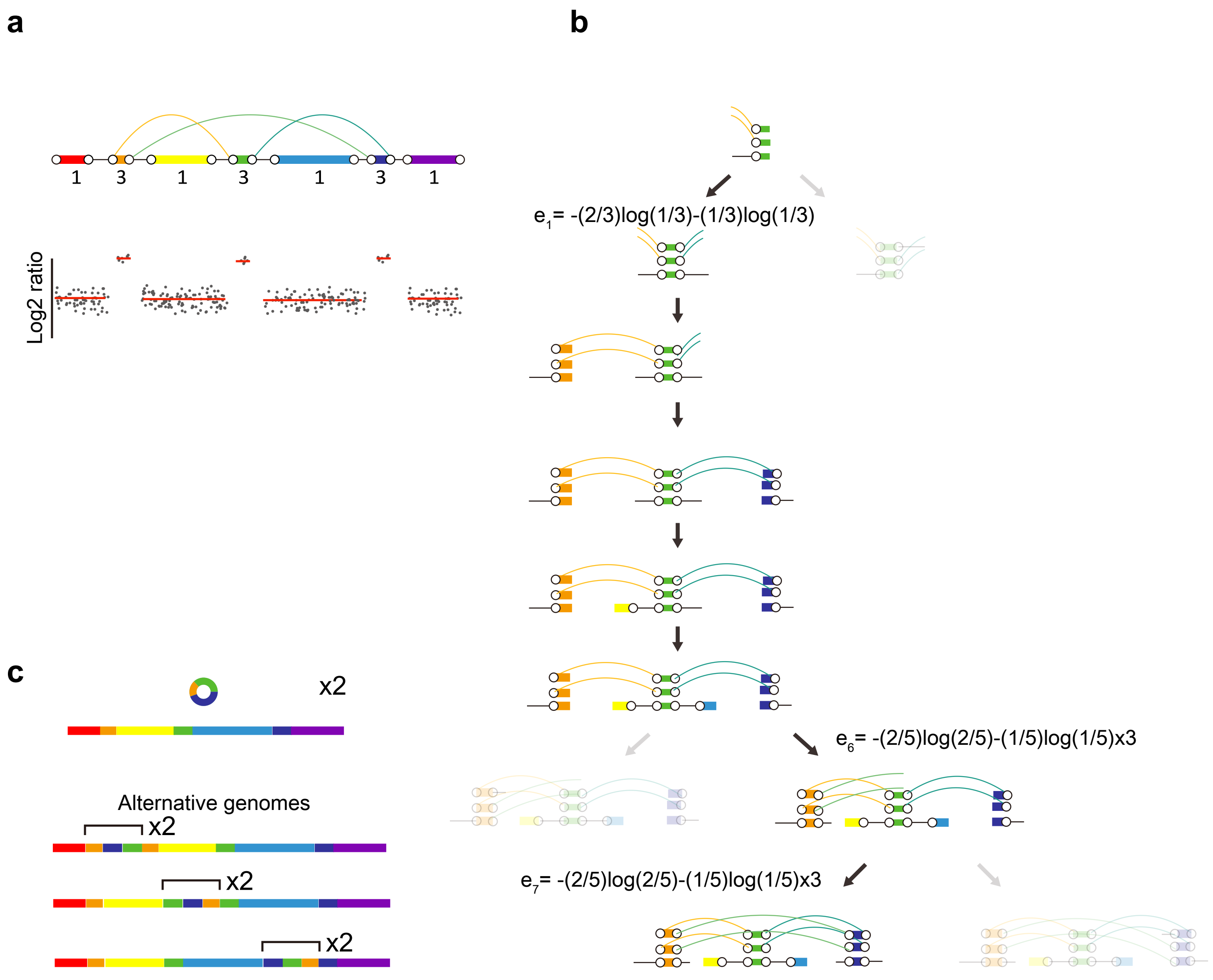

Supplementary Fig. 20: The multiway tree for Eulerian path enumerations and the minimum-entropy search. **a,** The copy number plot and breakpoint graph showing that focal amplifications are clustered by SV edges. **b,** The multiway tree, where each level represents edge pairing states of a head node or tail node in the breakpoint graph. The entropy of paths is measured at each level, and paths with minimum entropy are prioritised. **c,** The prioritised solution obtained from the multiway tree. Here, a repetitive cycle is extracted from the minimum entropy search. Other alternative genomes exist depending on which breakpoint the cycle is incorporated into.

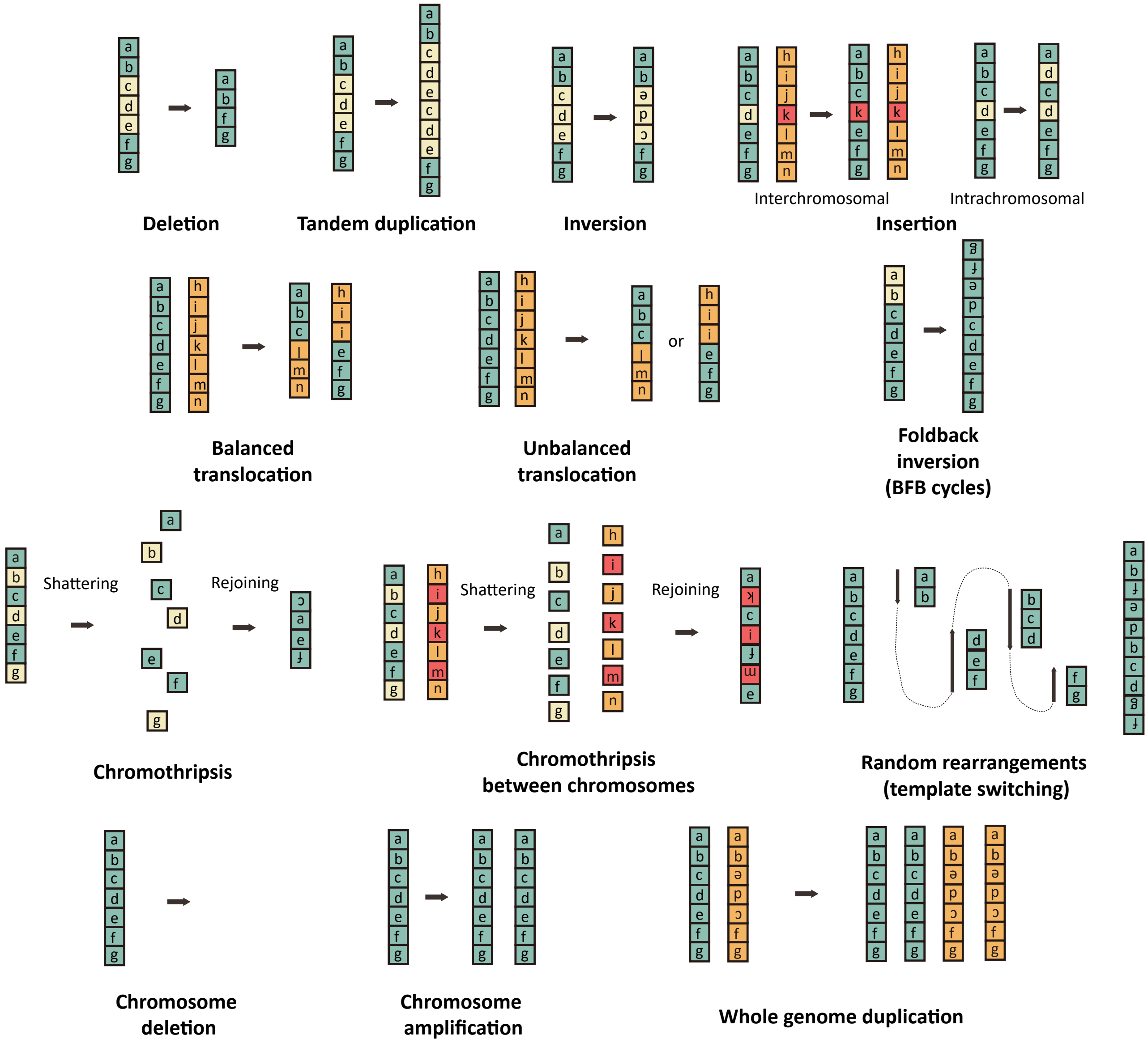

Supplementary Fig. 21: The simulation scheme. Operations that were used for cancer genome simulation. They include simple operations such as deletions, tandem duplications, and inversions. Insertions involve duplication of a segment from the same or a different chromosome and its insertion with or without loss of sequences with a random orientation. Balanced translocation involves exchange of two chromosomes without a loss or with a small deletion bridge, and unbalanced translocation involves exchange of two chromosomes, resulting in copy number changes. Fold-back inversion occurs at the end of the segment, generating an inverted duplication. Other complex rearrangements are generated through chromothripsis or template switching. Chromothripsis shatters a chromosome, and the shattered segments are rejoined randomly with the loss of unjoined segments. Template switching rearranges segments randomly, resulting in random copy number amplifications and deletions. Aneuploidy is generated through chromosomal deletions and amplifications, and whole-genome duplications are performed to generate triploid and tetraploid cancer genomes.

Table S1. Selected parameters/thresholds for variant callings

Method	Detection	Input SVs	Information	parameters/thresholds
InfoGenomeR	Total* SVs<br>Total SVCNs<br>Total CNAs<br>Total integer CNs<br>Total ASCNs<br>Total haplotype<br>Somatic SVs<br>Somatic SVCNs<br>Somatic CNAs<br>Somatic integer CNs<br>Somatic ASCNs<br>Somatic haplotype	DELLY2<br>Manta<br>novoBreak	Split/discordant reads<br>Unmapped reads<br>Read depths<br>SNP depths	Default<br>(the first-round lambda λ = 1, and the second-round lambda λ = 16)
DELLY2<br>(version 0.7.6)	Total SVs<br>somatic SVs		Split/discordant reads	The number of discordant reads<br>The number of soft-clipped reads<br>The mapping quality<br>PASS filter
Manta<br>(version 1.1.0)	Total SVs<br>somatic SVs		Split/discordant reads	The number of discordant reads<br>The number of soft-clipped reads
novoBreak<br>(version 1.1)	Total SVs<br>somatic SVs		Split/discordant reads<br>Unmapped reads	The number of discordant reads<br>The number of soft-clipped reads<br>The mapping quality
Weaver<br>(version 0.21)	Total SVs<br>Total SVCNs<br>Total CNAs<br>Total integer CNs<br>Total ASCNs<br>Total haplotype	The internal SV caller of Weaver	Split/discordant reads<br>Read depths or SNP depths	Default (as recommended)
CREST<br>(version 2.0)<br>+CONSERTING<br>(version 1.0)	Somatic SVs<br>Somatic CNAs	CREST	Split reads<br>Read depths	Default (as recommended)
CREST<br>(version 2.0)	Total SVs<br>somatic SVs		Split reads	The number of soft-clipped reads
BIC-seq2<br>(version 0.7.2)	Total CNAs<br>Somatic CNAs		Read depths	The lambda λ
BIC-seq2<br>(version 0.7.2)<br>+ABSOLUTE<br>(version 1.0.6)	Total integer CNs<br>Somatic integer CNs		Read depths	The sigma p
JaBbA<br>(version 0.0.0.9000)	Total SVs<br>Total SVCNs<br>Total CNAs<br>Total integer CNs<br>Somatic SVs<br>Somatic SVCNs<br>Somatic CNAs<br>Somatic integer CNs	DELLY2<br>Manta<br>novoBreak	Split/discordant reads<br>Unmapped reads<br>Read depths	The purity and ploidy target value<br>The fragCounter bin size (200bp or 1000bp)<br>The slack penalty (20, 100, 1000, or 2000)

* "Total" means no separation between germline and somatic variants.

Table S2. Performance metrics.

Categories	True prediction
SVs	The SV orientation is matched with the true SV orientation.<br>The breakpoints of the SV are matched with the true SV breakpoints within 100 bp.
SVCNs	The SV orientation is matched with the true SV orientation.<br>The breakpoints of the SV are matched with the true SV breakpoints within 100 bp.<br>The copy number of the SV is matched with the true copy number of the SV.
CNA breakpoints	The CNA breakpoint (up or down) is matched with the true breakpoint within 10kb.
Integer CNs	The segment is corroborated* with the true segment (90%).
ASCNs	The allelic segment is corroborated with the true allele segment (90%).
Haplotype	The inferred haplotype is matched with the true haplotype.<br>The switch error rate is measured as the proportion of switches of 1000 Genomes Project SNPs between the inferred and true haplotype.

* Corroboration means that the 90% of the segment has the same integer CN/ASCN with the true segment).

Table S3. The lung cancer cell lines and HeLa cell line.

	H292			InfoGenomeR prediction			
Chr	Karyotype*	TRA	Chr ends	Karyotype*	TRA	Chr ends	Unrecalled translocations
1	1<br>der(1)t(1;16)	t(1;16)	1pter x1<br>1qter x1<br>16qter x2	1<br>der(1)t(1;16)	t(1;16)	1pter x1<br>1qter x1<br>16qter x2	
2	2x2		2pter x2<br>2qter x2	2x2		2pter x2<br>2qter x2	
3	3x2		3pter x2<br>3qter x2	3x2		3pter x2<br>3qter x2	
4	4x2		4pter x2<br>4qter x2	4x2		4pter x2<br>4qter x2	
5	5<br>der(5)t(5;13)<br>i(5p)	t(5;13)	5pter x3<br>5qter x2<br>13qter x1	5x2<br>13<br>5pter->5cent x2		5pter x4<br>5cent x2<br>5qter x2<br>13pter x1<br>13qter x1	t(5;13)(pter;cent)
6	der(6)t(6;7)<br>der(6)t(6;8)	t(6;7)<br>t(6;8)	6pter x1<br>6qter x1<br>7qter x1<br>8qter x1	6<br>7pter->7cent<br>der(6)t(6;8)	t(6;8)	6pter x2<br>6qter x1<br>7pter x1<br>7qter x1<br>8qter x1	t(6;7)(pter;cent)
7	7<br>i(7p)		7pter x3<br>7qter x1	7x2		7pter x2<br>7qter x2	
8	8		8pter x1<br>8qter x1	8		8pter x1<br>8qter x1	
9	9x2		9pter x2<br>9qter x2	9x2		9pter x2<br>9qter x2	
10	10x2		10pter x2<br>10qter x2	10x2		10pter x2<br>10qter x2	
11	11x2<br>der(11)t(11;19)(<br>11p?->19pter)	t(11;19)	11pter x2<br>11p? x1<br>11qter x2<br>19pter x1	11x2<br>der(11)t(11;19)	t(11;19)	11pter x3<br>11qter x2<br>19pter x1	
12	12x2		12pter x2<br>12qter x2	12x2		12pter x2<br>12qter x2	
13	13<br>der(13)t(11;13)	t(11;13)	13pter x2<br>13qter x1<br>11pter x1	13		13pter x1<br>13qter x1	t(11;13)(cent;cent)
14	14x2		14pter x2<br>14qter x2	14x2		14pter x2<br>14qter x2	
15	15x2		15pter x2<br>15qter x2	15x2		15pter x2<br>15qter x2	
16	16<br>der(16)t(1;16)	t(1;16)	16pter x2<br>16qter x1<br>1qter x1	16<br>der(16)t(1;16)	t(1;16)	16pter x2<br>16qter x1<br>1qter x1	
17	17x2		17pter x2<br>17qter x2	17x2		17pter x2<br>17qter x2	
18	18x2		18pter x2<br>18qter x2	18x2		18pter x2<br>18qter x2	
19	19<br>der(19)t(11;19)	t(11;19)	19pter x1<br>19qter x2<br>11qter x1	19<br>der(19)t(11;19)<br>19q13->19qter	t(11;19)	19pter x1<br>19q13 x1<br>19qter x3<br>11qter x1	

20	20<br>i(20q)		20pter x1<br>20qter x3	20<br>20cent->20q x2		20pter x1<br>20cent x2<br>20qter x3		
21	21x2		21pter x2<br>21qter x2	21x2		21pter x2<br>21qter x2		
22	22		22pter x1<br>22qter x1	22		22pter x1<br>22qter x1		
X	Xx2		Xpter x2<br>Xqter x2	Xx2		Xpter x2<br>Xqter x2		

	A549			InfoGenomeR prediction			
Chr	Karyotype	TRA	Chr ends	Karyotype	TRA	Chr ends	Unrecalled translocations
1	1x2		1pter x2<br>1qter x2	1x2		1pter x2<br>1qter x2	
2	2x3<br>2cent->2qter		2pter x4<br>2cent x1<br>2qter x3	2x3<br>2cent->2qter		2pter x4<br>2cent x1<br>2qter x3	
3	3x2<br>der(3)t(3;20)	t(3;20)	3pter x3<br>3qter x2<br>20qter x1	3x2<br>3p14->3qter<br>20pter->20cent		3pter x3<br>3qter x2<br>3p14 x1<br>20pter x1<br>20cent x1	t(3;20)(p14;cent)
4	4x2		4pter x2<br>4qter x2	4x2		4pter x2<br>4qter x2	
5	5x3		5pter x3<br>5qter x3	5x3		5pter x3<br>5qter x3	
6	6<br>der(6)t(1;6)	t(1;6)	6pter x1<br>6qter x2<br>1qter x1	6x2<br>1cent->1qter		6pter x2<br>6qter x2<br>1cent x1<br>1qter x1	t(1;6)(cent;pter)
7	7x3		7pter x3<br>7qter x3	7x3		7pter x3<br>7qter x3	
8	8x3		8pter x3<br>8qter x3	8x3<br>8q24->8qter		8pter x3<br>8qter x4<br>8q24 x1	
9	9x3		9pter x3<br>9qter x3	9x3		9pter x3<br>9qter x3	
10	10x3		10pter x3<br>10qter x3	10x3		10pter x3<br>10qter x3	
11	11x2<br>der(11)t(8;11)	t(8;11)	11pter x3<br>11qter x2<br>8qter x1	11x2<br>der(11)t(8;11)	t(8;11)	11pter x3<br>11qter x2<br>8qter x1	
12	12x3		12pter x3<br>12qter x3	12x3		12pter x3<br>12qter x3	
13	13x2		13pter x2<br>13qter x2	13x2		13pter x2<br>13qter x2	
14	14x3		14pter x3<br>14qter x3	14x3		14pter x3<br>14qter x3	
15	15x2		15pter x2<br>15qter x2	15x2		15pter x2<br>15qter x2	
16	16x3		16pter x3<br>16qter x3	16x3		16pter x3<br>16qter x3	
17	17x4		17pter x4<br>17qter x4	17x4		17pter x4<br>17qter x4	

18	18x2		18pter x2<br>18qter x2	18x2		18pter x2<br>18qter x2	
19	19x2<br>der(19)t(15;19)x2	t(15;19)	19pter x2<br>19qter x4<br>15qter x2	19x2<br>der(19)t(15;19)x2	t(15;19)	19pter x2<br>19qter x4<br>15qter x2	
20	20x3		20pter x3<br>20qter x3	20x3		20pter x3<br>20qter x3	
21	21x2		21pter x2<br>21qter x2	21x2		21pter x2<br>21qter x2	
22	22x2		22pter x2<br>22qter x2	22x2		22pter x2<br>22qter x2	
X	Xx2		Xpter x2<br>Xqter x2	Xx2		Xpter x2<br>Xqter x2	

	H226			InfoGenomeR prediction			
Chr	Karyotype	TRA	Chr ends	Karyotype	TRA	Chr ends	Unrecalled translocations
1	1x4		1pter x4<br>1qter x4	1x4		1pter x4<br>1qter x4	
2	2x4		2pter x4<br>2qter x4	2x4		2pter x4<br>2qter x4	
3	3x4		3pter x4<br>3qter x4	3x4		3pter x4<br>3qter x4	
4	4x3		4pter x3<br>4qter x3	4x3		4pter x3<br>4qter x3	
5	5x4		5pter x4<br>5qter x4	5x4		5pter x4<br>5qter x4	
6	6x4		6pter x4<br>6qter x4	6x4		6pter x4<br>6qter x4	
7	7x4		7pter x4<br>7qter x4	7x4		7pter x4<br>7qter x4	
8	8x2<br>der(8)t(8;19)x2	t(8;19)	8pter x4<br>8qter x2<br>19pter x2	8x2<br>der(8)t(8;19)x2	t(8;19)	8pter x4<br>8qter x2<br>19pter x2	
9	9<br>der(9)t(9;20)x2	t(9;20)	9pter x2<br>9qter x4<br>20qter x2	9pter->9p21 x2<br>9q13->9qter x2<br>der(9)t(9;20)x2	t(9;20)	9pter x2<br>9p21 x2<br>9q13 x2<br>9qter x4<br>20qter x2	
10	der(10)t(7;10)x2<br>der(10)t(10;15)x2	t(7;10)<br>t(10;15)	10pter x2<br>7pter x2<br>15qter x2<br>10qter x2	der(10)t(7;10)x2<br>der(10)t(10;15)x2	t(7;10)<br>t(10;15)	10pter x2<br>7pter x2<br>15qter x2<br>10qter x2	
11	11x4		11prer x4<br>11qter x4	11x4		11pter x4<br>11qter x4	
12	12x2<br>der(12)t(12;15)x3	t(12;15)	12pter x2<br>12qter x5<br>15qter x3	12x4<br>12cent->12q21 x2		12pter x4<br>12cent x2<br>12q21 x2<br>12qter x4	(12;15)
13	13x2<br>der(13)t(13;?) x2	t(13;?)	13pter x2<br>13qter x4<br>? x2	13x2<br>13q13->13qter x2		13pter x2<br>13q13 x2<br>13qter x4	t(13:?)
14	14x2<br>der(14)t(14;?) x2	t(14;?)	14pter x2<br>14qter x4<br>? x2	14x3<br>der(14)t(12;14)	t(12;14)	14pter x4<br>12pter x1<br>14qter x3	t(14;?)
15	der(15)t(15;?)	t(15:?)	15pter x2<br>? x2	15x2		15pter x2<br>15qter x2	t(15;?)
16	16x4		16pter x4<br>16qter x4	16x4		16pter x4<br>16qter x4	
17	17x4		17pter x4<br>17qter x4	17x4		17pter x4<br>17qter x4	
18	18x2<br>18pter->18?x2		18pter x4<br>18qter x2<br>18? x2	18x2<br>18pter->18q12 x2		18pter x4<br>18qter x2<br>18q12 x2	

19	19x2<br>der(19)t(8;19;21)x2	t(8;19)<br>t(19;21)	19pter x2<br>8qter x2<br>19qter x2<br>21 qter x2	19x2<br>der(19)t(8;19)x2<br>19q13->19qter x2<br>21 x2	t(8;19)	19pter x2<br>8qter x2<br>19q13 x2<br>19qter x6<br>21pter x2<br>21qter x2	t(19;21)(qter;pter)
20	der(20)t(9;20)x2<br>der(20)t(20;21)x2	t(9;20)<br>t(20;21)	20pter x2<br>9pter x2<br>20qter x2<br>21qter x2	der(20)t(9;20)x2<br>der(20)t(20;21)x2	t(9;20)<br>t(20;21)	20pter x2<br>9pter x2<br>20qter x2<br>21qter x2	
21							
22	22x2		22pter x2<br>22qter x2	22x2		22pter x2<br>22qter x2	
X	Xx2		Xpter x2<br>Xqter x2	Xx2		Xpter x2<br>Xqter x2	

	HeLa			InfoGenomeR prediction			
Chr	Karyotype	TRA	Chr ends	Karyotype	TRA	Chr ends	Unrecalled translocations
1	1x2<br>der(1)t(1;3)<br>der(1)t(1;5)<br>der(1)t(1;6;7;14)(6pter-><br>7?)	t(1;3)<br>t(1;5)<br>t(1;6)<br>t(7;14)<br>t(1;14)	1pter x2<br>1qter x4<br>3qter x1<br>6pter x1<br>7? x1	1x2<br>der(1)t(1;3)<br>1p35->1qter<br>der(1)t(1;6)(1cent-><br>6pter)	t(1;3)<br>t(1;6)	1pter x2<br>1p35 x1<br>1cent x1<br>1qter x4<br>3qter x1<br>6pter x1	t(1;5)(cent;cent)<br>t(1;14)<br>t(7;14)
2	2x2<br>der(2)t(2;5;13)<br>der(2)t(2;5;X)	t(2;5)<br>t(2;13)<br>t(2;X)	2pter x2<br>2qter x2<br>5pter x2<br>13qter x1<br>Xpter x1	2x2<br>der(2)t(2;5;X)<br>13	t(2;5)<br>t(2;X)	2pter x2<br>2qter x2<br>Xpter x1<br>5pter x1<br>13pter x1<br>13qter x1	t(2;13)
3	3<br>der(3)t(3;12)<br>der(3)t(3;14)	t(3;12)<br>t(3;14)	3pter x2<br>3qter x2<br>12pter x1<br>14qter x1	3<br>der(3)t(3;12)<br>der(3)t(3;6)(3pter-><br>6p12)<br>14	t(3;12)	3pter x2<br>3qter x2<br>12pter x1<br>6p12 x1<br>14pter x1<br>14qter x1	t(3;14)(cent;pter)
4	4x2		4pter x2<br>4qter x2	4x2		4pter x2<br>4qter x2	
5	del(5)x2<br>der(5)t(3;5)<br>der(5)t(5;20)<br>5per->5cent	t(3;5)<br>t(5;20)	5pter x4<br>5cent x1<br>5qter x3<br>3pter x1<br>20qter x1	5<br>del(5)<br>5cent->5qter<br>5pter->5cent x5<br>3pter->3p14<br>20cent->20qter		5pter x7<br>5cent x6<br>5qter x3<br>3pter x1<br>3p14x1<br>20cent x1<br>20qter x1	t(3;5)(p14;cent)<br>t(5;20)(cent;cent)
6	6x2<br>der(6)t(6;19)	t(6;19)	6pter x3<br>6qter x2<br>19pter x1	6x2<br>der(6)t(6;19)	t(6;19)	6pter x3<br>6qter x2<br>19pter x1	
7	7x2<br>der(7)t(7;21)(7qter-><br>21?)	t(7;21)	7pter x2<br>7qter x3<br>21? x1	7x3		7pter x3<br>7qter x3	t(7;21)
8	8x2<br>8cent->8qter		8pter x2<br>8cent x1<br>8qter x3	8x2<br>8cent->8qter		8pter x2<br>8cent x1<br>8qter x3	
9	9x3		9pter x3<br>9qter x3	9x3		9pter x3<br>9qter x3	
10	10x3		10pter x3<br>10qter x3	10x3		10pter x3<br>10qter x3	
11	11x2<br>der(11)t(5;7;11)(7q?-><br>5pter)	t(7;11)<br>t(5;11)	11pter x2<br>11qter x2<br>7q? X1<br>5pter x1	11x2<br>der(11)t(7;11)(7q33-><br>11q14)<br>5pter->5cent x1	t(7;11)	11pter x2<br>11qter x2<br>7q33 x1<br>11q14 x1<br>5cent x1<br>5pter x1	t(5;11)(cent;q14)
12	12x3		12pter x3<br>12qter x3	12x3		12pter x3<br>12qter x3	

13	13<br>der(13)t(5;13;X)(5?->X?)	t(5;13)<br>t(13;X)	13pter x1<br>13qter x1<br>5? x1<br>X? x1	13x2		13pter x2<br>13qter x2	t(5;13)<br>t(13;X)
14	14		14pter x1<br>14qter x1	14		14pter x1<br>14qter x1	
15	15x3		15pter x3<br>15qter x3	15x3		15pter x3<br>15qter x3	
16	16x2		16pter x2<br>16qter x2	16x3<br>16pter->16p13<br>16q24->16qter		16pter x4<br>16p13 x1<br>16q24 x1<br>16qter x4	
17	17x3		17pter x3<br>17qter x3	17x3<br>17q24->17qter		17pter x3<br>17q24 x1<br>17qter x4	
18	18x2		18pter x2<br>18qter x2	18x2<br>18pter->18p11		18pter x3<br>18p11 x1<br>18qter x2	
19	19<br>19pter->19q13<br>der(19)t(13;19)	t(13;19)	19pter x2<br>19q13 x1<br>19qter x2	19<br>19pter->19q13<br>der(19)t(13;19)	t(13;19)	19pter x2<br>19q13 x1<br>19qter x2	
20	20x2		20pter x2<br>20qter x3	20x2		20pter x2<br>20qter x2	
21	21x2		21pter x2<br>21qter x2	21x2		21pter x2<br>21qter x2	
22	22<br>der(22)t(8;22)	t(8;22)	22pter x2<br>22qter x1<br>8qter x1	22x3<br>8q24->8qter		22pter x3<br>22qter x3<br>8q24 x1<br>8qter x1	t(8;22)(q24;22qter)
X	Xx2		Xpter x2<br>Xqter x2	Xx2<br>Xpter->Xcent		Xpter x3<br>Xcent x1<br>Xqter x2	

* The karyotypes of lung cancer cell lines were annotated by a manual inspection of m-FISH karyotypes from the lung cancer cell line paper[25].

* The karyotype of the HeLa cell line was annotated by a manual inspection of the m-FISH karyotype from the HeLa cell line paper[9].

* der(chr): a derivative chromosome generated from multiple chromosomes with the centromere of chr.

* t(chr1;chr2): a translocation between chr1 and chr2.

* (start->end): Indicating the start and end of a chromosome (or a segment).

* pter and qter: the p-terminal and q-terimal of a chromosomal arm.

* cent: the centromere of a chromosome.

* i(chr): isochromosome composed of p-arm or q-arm of chr.

Table S4. SV callings.

Requirements

Files	hg19.fa: the human reference sequence.<br>tumor.bam: an alignment file of a tumor.<br>normal.bam: an alignment file of a normal.<br>samples.tsv: a sample description file required for DELLY2.<br>hg19.bam: an alignment file of random reads from the hg19 (recommendation for the novoBreak total mode).	

DELLY2 (version 0.7.6)

Modes	Commands	Preprocessing
total	delly_v0.7.6 call -t DEL -g hg19.fa -o DEL.bcf tumor.bam	≥ 3 discordant and split reads<br>mapping quality ≥ 20<br>"PASS" filter
	delly_v0.7.6 call -t INV -g hg19.fa -o INV.bcf tumor.bam	
	delly_v0.7.6 call -t DUP -g hg19.fa -o DUP.bcf tumor.bam	
	delly_v0.7.6 call -t TRA -g hg19.fa -o TRA.bcf tumor.bam	
somatic	delly_v0.7.6 call -t DEL -g hg19.fa -o somatic_pre_DEL.bcf tumor.bam normal.bam \\<br>delly_v0.7.6 filter -t DEL -f somatic -o somatic_DEL.bcf -s samples.tsv somatic_pre_DEL.bcf -a 0 -m 100	≥ 3 discordant and split reads<br>mapping quality ≥20<br>"PASS" filter
	delly_v0.7.6 call -t INV -g hg19.fa -o somatic_pre_INV.bcf tumor.bam normal.bam \\<br>delly_v0.7.6 filter -t INV -f somatic -o somatic_INV.bcf -s samples.tsv somatic_pre_INV.bcf -a 0 -m 100	
	delly_v0.7.6 call -t DUP -g hg19.fa -o somatic_pre_DUP.bcf tumor.bam normal.bam \\<br>delly_v0.7.6 filter -t DUP -f somatic -o somatic_DUP.bcf -s samples.tsv somatic_pre_DUP.bcf -a 0 -m 100	
	delly_v0.7.6 call -t TRA -g hg19.fa -o somatic_pre_TRA.bcf tumor.bam normal.bam \\<br>delly_v0.7.6 filter -t TRA -f somatic -o somatic_TRA.bcf -s samples.tsv somatic_pre_TRA.bcf -a 0 -m 100	

Manta (version 1.1.0)

Modes	Commands	Preprocessing
total	configManta.py --tumorBam tumor.bam --referenceFasta hg19.fa --runDir manta \\<br>runWorflow.py -m local -j 1	≥ 3 discordant and split reads<br>"PASS" filter
somatic	configManta.py --normalBam normal.bam --tumorBam tumor.bam --referenceFasta hg19.fa --runDir manta_somatic \\<br>runWorflow.py -m local -j 1	≥ 3 discordant and split reads<br>"PASS" filter

novoBreak (version 1.1.3)

Modes	Commands	Preprocessing
total	run_novoBreak.sh novoBreak_distribution_v1.1.3rc hg19.fa tumor.bam hg19.bam 8 novobreak	≥ 3 discordant and split reads<br>mapping quality ≥ 20
somatic	run_novoBreak.sh novoBreak_distribution_v1.1.3rc hg19.fa tumor.bam normal.bam 8 novobreak	≥ 3 discordant and split reads<br>mapping quality ≥ 20

Table S5. Metrics for private and shared SVs.

**Primary**		**Metastasis/Relapse**		**Prediction**	**SV class**
**Discordant /split reads**	**μ(SV edge) > 0**	**Discordant /split reads**	**μ(SV edge) > 0**		
X	X	O	X	false SV	
X	X	O	O	true SV	private SV to metastasis/relapse
X	O	O	**X**	**false SV**	**candidate shared SV***
X	O	O	O	true SV	shared SV
O	X	X	X	false SV	
O	**X**	X	O	**false SV**	**candidate shared SV***
O	O	X	X	true SV	private SV to primary
O	O	X	O	true SV	shared SV
O	X	O	X	false SV	
O	**X**	O	O	**false SV**	**candidate shared SV***
O	O	O	**X**	**false SV**	**candidate shared SV***
O	O	O	O	true SV	shared SV

*It is checked whether μ(candidate SV edge)>0 by reoptimizing the breakpoint graph.

Table S6. SV simulation.

Event	Type (operation)	Proportion	Simulation method	Beta dist. alpha	Beta dist. beta
Germline	Deletion	0.686	generation	0.460	62.910
	Inversion	0.009	generation	0.523	2.667
	Duplication	0.004	generation	0.896	11.244
	Insertion (numt)	0.001	selection		
	Insertion (alu)	0.230	selection		
	Insertion (L1)	0.055	selection		
	Insertion (SVA)	0.015	selection		
Somatic	Deletion	0.173	generation	0.266	1.905
	Inversion	0.009	generation	0.429	1.263
	Duplication	0.174	generation	0.263	2.686
	Insertion	0.072	generation	0.428	1.979
	Intrachromosomal rearrangement (foldback inversion, chromothripsis, template switching)	0.397	generation		
	Interchromosomal rearrangement (balanced/unbalanced translocation, chromothripsis, template switching)	0.176	generation		

Supplementary References

1. Zook, J. M. *et al.* Extensive sequencing of seven human genomes to characterize benchmark reference materials. *Scientific data* **3**, 160025 (2016).

2. Chaisson, M. J. *et al.* Multi-platform discovery of haplotype-resolved structural variation in human genomes. *Nat. Commun.* **10** (2019).

3. Bartenhagen, C. & Dugas, M. Rsvsim: an r/bioconductor package for the simulation of structural variations. *Bioinformatics* **29**, 1679–1681 (2013).

4. Monlong, J. *et al.* Human copy number variants are enriched in regions of low mappability. *Nucleic Acids Res* **46**, 7236–7249 (2018).

5. Yang, L. *et al.* Diverse mechanisms of somatic structural variations in human cancer genomes. *Cell* **153**, 919–929 (2013).

6. Aran, D., Sirota, M. & Butte, A. J. Systematic pan-cancer analysis of tumour purity. *Nat. Commun.* **6**, 8971 (2015).

7. Huang, W., Li, L., Myers, J. R. & Marth, G. T. Art: a next-generation sequencing read simulator. *Bioinformatics* **28**, 593–594 (2011).

8. Li, H. Aligning sequence reads, clone sequences and assembly contigs with bwa-mem. arXiv (2013). Https://arxiv.org/abs/1303.3997.

9. Landry, J. J. *et al.* The genomic and transcriptomic landscape of a HeLa cell line. *G3 (Bethesda)* **3**, 1213–1224 (2013).

10. Shen, M. M. Chromoplexy: a new category of complex rearrangements in the cancer genome. *Cancer Cell* **23**, 567–569 (2013).

11. Deshpande, V. *et al.* Exploring the landscape of focal amplifications in cancer using ampliconarchitect. *Nat. Commun.* **10** (2019).

12. Carroll, S. *et al.* Double minute chromosomes can be produced from precursors derived from a chromosomal deletion. *Mol. Cell. Biol.* **8**, 1525–1533 (1988).

13. Korbel, J. O. & Campbell, P. J. Criteria for inference of chromothripsis in cancer genomes. *Cell* **152**, 1226–1236 (2013).

14. Cortés-Ciriano, I. *et al.* Comprehensive analysis of chromothripsis in 2,658 human cancers using whole-genome sequencing. *Nature Genet.* **52**, 331–341 (2020).

15. Knutsen, T. *et al.* The interactive online sky/m-fish & cgh database and the entrez cancer chromosomes search database: linkage of chromosomal aberrations with the genome sequence. *Genes, Chromosomes and Cancer* **44**, 52–64 (2005).

16. Killcoyne, S. & del Sol, A. Identification of large-scale genomic variation in cancer genomes using in silico reference models. *Nucleic Acids Res.* **44**, e5–e5 (2015).

17. Li, H., Ruan, J. & Durbin, R. Mapping short dna sequencing reads and calling variants using mapping quality scores. *Genome Res.* **18**, 1851–1858 (2008).

18. Kent, W. J. Blat-the blast-like alignment tool. *Genome Res.* **12**, 656–664 (2002).

19. Li, Y., Zhou, S., Schwartz, D. C. & Ma, J. Allele-specific quantification of structural variations in cancer genomes. *Cell Syst.* **3**, 21–34 (2016).

20. Aganezov, S. & Raphael, B. J. Reconstruction of clone-and haplotype-specific cancer genome karyotypes from bulk tumor samples. *Genome Res.* **30**, 1274–1290 (2020).

21. Carvalho, C. M. & Lupski, J. R. Mechanisms underlying structural variant formation in genomic disorders. *Nat. Rev. Genet.* **17**, 224 (2016).

22. Altschul, S. F., Gish, W., Miller, W., Myers, E. W. & Lipman, D. J. Basic local alignment search tool. *J. Mol. Biol.* **215**, 403–410 (1990).

23. Browning, S. R. & Browning, B. L. Rapid and accurate haplotype phasing and missing-data inference for whole-genome association studies by use of localized haplotype clustering. *Am. J. Hum. Genet.* **81**, 1084–1097 (2007).

24. Kholilurrohman, M. & Minato, S.-i. An efficient algorithm for enumerating eulerian paths. *Hokkaido University, Division of Computer Science, TCS Technicial Reports* TCS–TR–A–14–77 (2014).

25. Liu, J. *et al.* Genome and transcriptome sequencing of lung cancers reveal diverse mutational and splicing events. *Genome res.* **22**, 2315–2327 (2012).
