# Supplementary material for: Integrative reconstruction of cancer genome karyotypes using InfoGenomeR
Source: Nat Commun. 2021 Apr 29;12:2467. doi: 10.1038/s41467-021-22671-6 (PMC8085216; doi:10.1038/s41467-021-22671-6)
Supplement: Supplementary file 3 — Reporting Summary [file 41467_2021_22671_MOESM3_ESM.pdf]

nature research

Corresponding author(s): Hyunju Lee

Last updated by author(s): Feb 25, 2021

Reporting Summary

Nature Research wishes to improve the reproducibility of the work that we publish. This form provides structure for consistency and transparency in reporting. For further information on Nature Research policies, see our Editorial Policies and the Editorial Policy Checklist.

Statistics

For all statistical analyses, confirm that the following items are present in the figure legend, table legend, main text, or Methods section.

n/a	Confirmed	
☐	☒	The exact sample size (n) for each experimental group/condition, given as a discrete number and unit of measurement
☒	☐	A statement on whether measurements were taken from distinct samples or whether the same sample was measured repeatedly
☒	☐	The statistical test(s) used AND whether they are one- or two-sided<br>*Only common tests should be described solely by name; describe more complex techniques in the Methods section.*
☒	☐	A description of all covariates tested
☒	☐	A description of any assumptions or corrections, such as tests of normality and adjustment for multiple comparisons
☒	☐	A full description of the statistical parameters including central tendency (e.g. means) or other basic estimates (e.g. regression coefficient) AND variation (e.g. standard deviation) or associated estimates of uncertainty (e.g. confidence intervals)
☒	☐	For null hypothesis testing, the test statistic (e.g. F, t, r) with confidence intervals, effect sizes, degrees of freedom and P value noted<br>*Give P values as exact values whenever suitable.*
☒	☐	For Bayesian analysis, information on the choice of priors and Markov chain Monte Carlo settings
☒	☐	For hierarchical and complex designs, identification of the appropriate level for tests and full reporting of outcomes
☒	☐	Estimates of effect sizes (e.g. Cohen's d, Pearson's r), indicating how they were calculated

Our web collection on statistics for biologists contains articles on many of the points above.

Software and code

Policy information about availability of computer code

Data collection	The SRA Toolkit (version 2.8.2) was used to download the WGS and RNA-seq FASTQ files of the HeLa cell line and the WGS BAM files of lung cancer cell lines. The GDC client (version 1.2.0) was used to download the WGS BAM files of TCGA samples. The EGA client (version 2.2.2) was used to download the WGS BAM files of metastatic breast cancers.
Data analysis	Preprocessing<br>ART (version 2.5.8) was used for WGS-read simulation. BWA-MEM (version 0.7.15) was used for mapping WGS reads. SAMtools (version 1.3) was used for analyzing BAM files. HISAT2 (version 2.1.0) and CuffLinks (version 2.2.1) were used for mapping RNA-seq reads and quantifying gene expression values.<br><br>Integrative framework for genome reconsruction (InfoGenomeR)<br>R (version 3.4.3) was used for breakpoint graph construction. DELLY2 (version 0.7.6), Manta (version 1.1.0), and novoBreak (version 1.1) were used for SV detection. BIC-seq2 (version 0.7.2) was used for CNA detection. ABSOLUTE (version 1.0.6) was used for purity, ploidy, and integer copy number estimation. JaBbA (version 0.0.0.9000), Weaver (version 0.21), CREST (version 2.0), and CONSERTING (version 1.0) were used for comparison study of variant detection. the lpSolveAPI R package (version 5.5.2.0.17) was used for integer programming. BLAT (version 36) was used for mapping non-properly paired reads. BCFtools (version 1.3) was for SNP calling. We implemented the EM algorithm using R. BLAST (version 2.2.30) was used for local alignment. We implemented the BEAGLE method using C++ (version 4.8.2) for haplotype phasing. We implemented the Eulerian path enumeration using C++. The source codes are available at https://github.com/dmcblab/InfoGenomeR.

For manuscripts utilizing custom algorithms or software that are central to the research but not yet described in published literature, software must be made available to editors and reviewers. We strongly encourage code deposition in a community repository (e.g. GitHub). See the Nature Research guidelines for submitting code & software for further information.

Data

Policy information about availability of data

All manuscripts must include a data availability statement. This statement should provide the following information, where applicable:

- Accession codes, unique identifiers, or web links for publicly available datasets
- A list of figures that have associated raw data
- A description of any restrictions on data availability

Data availability
WGS and RNA-seq data of the HeLa cell line are available in the database of Genotypes and Phenotypes (dbGaP; accession code No. phs000643.v10.p1) [https://www.ncbi.nlm.nih.gov/projects/gap/cgi-bin/study.cgi?study id=phs000643.v10.p1]. WGS data of lung cancer cell lines are available in the dbGaP (accession code phs000299.v2.p1) [https://www.ncbi.nlm.nih.gov/projects/gap/cgi-bin/study.cgi?study id=phs000299.v2.p1]. WGS data of TCGA samples (BRCAs, OVs, and GBMs) are available in the dbGaP (accession code phs000178.v11.p8) [https://www.ncbi.nlm.nih.gov/projects/gap/cgi-bin/study. cgi?study id=phs000178.v11.p8]. WGS data of relapsed or metastatic breast cancers are available in the EGA (accession code EGAD00001002696) [https://ega-archive.org/datasets/EGAD00001002696]. Simulated datasets from NA12878 for GRCh37 and GRCh38 are available in Zenodo [https://doi.org/10.5281/zenodo.4545666]. Simulated datasets from HG00732, NA19238, and HG00513 for GRCh37 are available in Zenodo [https://doi.org/10.5281/zenodo.4556315]. The remaining data are available within the Article and Supplementary Information, or from the authors upon request.

Field-specific reporting

Please select the one below that is the best fit for your research. If you are not sure, read the appropriate sections before making your selection.

☒ Life sciences ☐ Behavioural & social sciences ☐ Ecological, evolutionary & environmental sciences

For a reference copy of the document with all sections, see nature.com/documents/nr-reporting-summary-flat.pdf

Life sciences study design

All studies must disclose on these points even when the disclosure is negative.

Sample size	The sample sizes were derived from published datasets. The HeLa cell line and three lung cancer cell lines (H292, A549, and H226) were analyzed, of which karyotypes have been previously reported using multipliex fluorescence in situ hybridization. Breast invasive carcinoma (BRCA, n=90), Glioblastoma multiforme (GBM, n=37), and ovarian serous cystadenocarcinoma (OV, n=47) were analyzed (TCGA). The sample sizes (n=90, 37, and 47) were the number of all the WGS data available from TCGA at that time we downloaded for BRCA, GBM, and OV, respectively. Fifteen patients with metastatic/relapsed breast cancers were analyzed (EGAD00001002696). The samples size (n=15) was the number of patients except for two patients (see Data exclusions) from the entire dataset (n=17) of metastatic breast cancers that were previously reported.
Data exclusions	Two patients in metastatic/relapsed breast cancers (EGAD00001002696, n=17) were excluded because they had no label of primary, metastatic or relapse for WGS data. Fifteen patients (15/17) had the label of tumor lesions, and they were analyzed in this study.
Replication	This study analyses published datasets and replication was not relevant.
Randomization	This study analyses published datasets and randomization was not relevant.
Blinding	This study analyses published datasets and blinding was not relevant.

Reporting for specific materials, systems and methods

We require information from authors about some types of materials, experimental systems and methods used in many studies. Here, indicate whether each material, system or method listed is relevant to your study. If you are not sure if a list item applies to your research, read the appropriate section before selecting a response.

Materials & experimental systems

n/a	Involved in the study	
☒	☐	Antibodies
☒	☐	Eukaryotic cell lines
☒	☐	Palaeontology and archaeology
☒	☐	Animals and other organisms
☒	☐	Human research participants
☒	☐	Clinical data
☒	☐	Dual use research of concern

Methods

n/a	Involved in the study	
☒	☐	ChIP-seq
☒	☐	Flow cytometry
☒	☐	MRI-based neuroimaging
